# Assessing the reliability of medical resource demand models in the context of COVID-19

**DOI:** 10.1186/s12911-024-02726-6

**Published:** 2024-10-31

**Authors:** Kimberly Dautel, Ephraim Agyingi, Pras Pathmanathan

**Affiliations:** 1 School of Mathematics and Statistics, Rochester Institute of Technology, 84 Lomb Memorial Dr, Rochester, New York USA; 2 Division of Biomedical Physics, Office of Science and Engineering Laboratories, Center for Devices and Radiological Health, Food and Drug Administration, 10903 New Hampshire Ave, Silver Spring, Maryland USA

**Keywords:** Mathematical modeling, Epidemiology, Medical device, Validation

## Abstract

**Background:**

Numerous medical resource demand models have been created as tools for governments or hospitals, aiming to predict the need for crucial resources like ventilators, hospital beds, personal protective equipment (PPE), and diagnostic kits during crises such as the COVID-19 pandemic. However, the reliability of these demand models remains uncertain.

**Methods:**

Demand models typically consist of two main components: hospital use epidemiological models that predict hospitalizations or daily admissions, and a demand calculator that translates the outputs of the epidemiological model into predictions for resource usage. We conducted separate analyses to evaluate each of these components. In the first analysis, we validated various hospital use epidemiological models using a recent validation framework designed for epidemiological models. This allowed us to quantify the accuracy of the models in predicting critical aspects such as the date and magnitude of local COVID-19 peaks, among other factors. In the second analysis, we evaluated a range of demand calculators for ventilators, medical gowns, and COVID-19 test kits. To achieve this, we decoupled these demand calculators from the underlying epidemiological models and provided ground truth data for their inputs. This approach enabled a direct comparison of the demand calculators, comparing them against each other and actual usage data when available. The code is available at https://doi.org/10.5281/zenodo.13712387.

**Results:**

Performance varied greatly across the epidemiological models, with greater variability in COVID-19 hospital use predictions than for COVID-19 deaths as analyzed previously. Some models did not have any peaks. Among those that did, the models under-estimated date of peak approximately as often as they over-estimated, but were more likely to under-estimate magnitude of peak, with typical relative errors around 50%. Regarding demand calculator predictions, there was significant variability, including five-fold differences in predictions for gown models. Validation against actual or surrogate usage data illustrated the potential value of demand models while demonstrating their limitations.

**Conclusions:**

The emerging field of demand modeling holds promise in averting medical resource shortages during future public health emergencies. However, achieving this potential necessitates focused efforts on standardization, transparency, and rigorous model validation before placing reliance on demand models in critical public health decision-making.

**Supplementary Information:**

The online version contains supplementary material available at 10.1186/s12911-024-02726-6.

## Background

Forecasting medical resource demand is an essential operation to provide effective treatment of patients and protect healthcare workers when delivering care. Under normal hospital operations, little forecasting is needed as hospital administrators replenish orders for materials recently consumed. Furthermore, there will be additional unused resources available. However, during exceptional circumstances such as the COVID-19 pandemic, hospitals may not have been adequately prepared and may be strained for resources.

Various groups have developed quantitative medical resource demand models as tools that governments, local authorities, or hospitals could utilize for planning and resource allocation. These predictive demand models, for important resources such as ventilators, hospital beds, personal protective equipment (PPE), and diagnostic kits, are expected to play a key role in efforts to counter future supply chain shortages, such as in the Resilient Supply Chain Program recently initiated by FDA’s Center for Devices and Radiological Health [1]. A typical modeling workflow consists of running an epidemiological model to predict expected cases or hospitalizations in a region of interest, which are used to estimate the amount of a given resource needed, based on historical information about usage of the resource per patient and caregiver. However, the reliability of resource demand models is unclear and model evaluation efforts lag model development efforts. It is critically important to understand the predictive capability of resource demand models in advance of their use in future public health emergencies. Therefore, in this paper we conduct the first analysis, to our knowledge, evaluating the general reliability of medical resource demand models.

### Review of medical resource demand models

In this section we discuss some key medical resource demand models (henceforth, just ‘demand models’) developed in response to recent infectious disease outbreaks. Table 1 provides an overview of US demand models developed during the COVID-19 pandemic. See also [2] for an in depth review of recent demand models.

**Table 1:** Selected US resource demand models developed in response to the COVID-19 pandemic

Organization	Resources predicted	Region of interest	Inputs provided by user	Epidemiological model
WHO [3]	PPE, diagnostics, consumable medical supplies, biomedical equipment, and essential drugs	Countries	Cumulative COVID-19 cases at end of week (for manual input)	Option of Imperial College London SEIR model, SIR model, or manual input
University of Pennsylvania and Penn Medicine (Penn) [4]	PPE	Hospital or health system	COVID-19 patients hospitalized, in ICU, on ventilator, and new COVID-19 admissions (for manual input)	CHIME (COVID-19 Hospital Impact Model for Epidemics)
Maryland Emergency Management Agency [5]	PPE	Maryland statewide and countywide	COVID-19 patients that day and previous 24, 48, and 72 hours; total number of EMS response calls for the past 24 hours (for manual input)	Johns Hopkins University Applied Physics Laboratory (MD APL Model)
Rush University Medical Center [6]	Beds, ventilators, and PPE	States and territories	Unknown (as model inaccessible at time of writing)	7 different models
Wells et al. [7]	Ventilators	Nationwide	Initial infectious cases in each age group in Moghadas et al. model [8]	Age-structured dynamic model [8]
IHME [9]	Ventilators	US states	Data on confirmed COVID-19 deaths	Curve fitting statistical model projected death rates which from this estimated hospital service utilization using an individual-level microsimulation model
JHU [10]	PPE	US	COVID-19 deaths	None
CDC [11]	PPE	Healthcare facility	Suspected and confirmed COVID-19 patients, on hand and resupply each day	None
Virginia Tech [12]	PPE	Hospital	COVID-19/person under investigation patients	None
COVID Staffing Project [13]	PPE	Hospital	Number of patients hospitalized, in ICU, on ventilator	None
Children’s National Hospital and the George Washington University (CNH) [14]	PPE	Hospital	All patients in next 24 hours, level of conservation	None

During the largest Ebola outbreak in 2014, the US Centers for Disease Control and Prevention (CDC) produced a PPE calculator tool designed to help estimate the amount of PPE hospitals may need to manage the care of one patient hospitalized with Ebola [15]. The US Administration for Strategic Preparedness and Response (ASPR) developed the Hospital Personal Protective Equipment Planning Tool designed to help hospitals approximate minimum PPE needs during three types of infectious disease outbreaks: Ebola Virus Disease/Viral Hemorrhagic Fever (EVD/VHF), special respiratory pathogens such as Middle East Respiratory Syndrome/Severe Acute Respiratory Syndrome (MERS/SARS), and pandemic influenza [16]. Early during the COVID-19 pandemic the Johns Hopkins Bloomberg School of Public Health developed a tool which calculated the national PPE demand for COVID-19 [10]. The CDC Personal Protective Equipment Burn Rate Calculator was also developed and designed to help healthcare facilities plan and optimize the use of PPE for response to COVID-19 [11].

The WHO COVID-19 Essential Supplies Forecasting Tool [3] is noteworthy as it is the only model to provide a forecast for a multitude of countries and various types of medical resources. It was created to assist governments, partners, and other stakeholders in estimating possible needs for critical supplies to address the ongoing COVID-19 outbreak. The WHO tool outputs predictions for five categories of essential commodities: hygiene, personal protective equipment, diagnostics, drugs and consumables, and biomedical equipment for case management. The WHO model is the most complex model we encountered, accounting for patient severity, healthcare workforce, health infrastructure, testing capacity, and oxygen utilization.

Demand models are closely related to epidemiological models, with outputs from the latter often directly impacting the demand predictions. For example, the WHO COVID-19 Essential Supplies Forecasting Tool [3] provides the user with a choice among several epidemiological methods for forecasting COVID-19 cases, including an integration with Imperial College’s Susceptible-Exposed-Infectious-Removed (SEIR) model [17]. The underlying SEIR model is age-structured where the infectious class is divided into different stages reflecting progression through different disease severity pathways. Alternatively, there is an embedded SIR model option. McCabe et al. [18] integrates hospital capacity planning and epidemiological projections of COVID-19 patients to estimate the demand for and resultant spare capacity of ICU beds, staff and ventilators. This model also utilizes Imperial College’s SEIR model [17]. Wells et al. [7] projected the demand for ventilators at the peak of the COVID-19 outbreak in the USA by combining an age-structured dynamic model of SARS-CoV-2 transmission and current data [8]. This age-structured compartmental model incorporated a breakdown of infectious cases based on the severity of the infection and whether self-isolation was practiced. Additionally, there was a breakdown of hospitalizations based upon whether patients were in the intensive care unit or not.

Therefore, demand models can be conceptually broken into two distinct parts: the **epidemiological model** designed to explain and predict the spread of an infectious disease, and a post-processing component that we will refer to as the **demand calculator**. The demand calculator predicts the total number of medical resources needed based on the outputs of the epidemiological model, and other factors such as availability and number of patients and healthcare providers. Many COVID-19 related demand models have an epidemiological model component that predicts the number of COVID-19 hospitalizations, COVID-19 admissions, and/or COVID-19 patients in the ICU. However, other groups developed demand tools with no underlying epidemiological model, for which the user is required to input the number of COVID-19 hospitalizations, COVID-19 admissions, and/or COVID-19 patients in the ICU (e.g., JHU [10], Children’s National Hospital [14]). These types of demand models serve as just a demand calculator.

### Model-based decision making

Medical resource demand modeling is beneficial for healthcare resource and allocation, as improved forecasts strengthen the supply chain and the broader healthcare system. These models have the potential to support decision making by predicting the magnitude of medical resources required and when the resources are needed. An underestimation of hospitalizations can result in a shortage of medical resources necessary to operate. Consequently, healthcare workers may be put at increased risk of exposure and patients may be turned away if the hospital is unable to provide the appropriate amount of healthcare needed. On the other hand, an overestimation of hospitalizations in a given location may not have severe consequences, though it could lead to resources taken away from another hospital in need. Furthermore, the amount of overestimated product may lead to expired/wasted product. The timing of when supplies are delivered and available is crucial to providing proper medical care. Improved ordering decisions based on more precise forecasts would eventually increase product availability for customers and may even reduce waste through more effective supply chain management [19].

Specific examples of how demand models are used in practice are hard to ascertain. One example is [20], which describes how healthcare financial managers can use demand models to improve their organization’s performance and help deliver high-quality healthcare. Manufacturers across industries can use the forecast of medical resource demand models to adjust for rapid changes in demand. Health authorities and governments can use demand models to optimize the distribution of medical resources such as vaccines [21]. From healthcare organizations, medical suppliers, to manufacturers, there are many entities that could utilize medical resource demand modeling. As a result, these models could become a critical component of healthcare product management.

### Aims

The goals of this paper are to evaluate the reliability of medical resource demand models, identify key features that affect demand model accuracy, and provide recommendations for advancing the field of demand modeling.

Given that there are many possibilities for the epidemiological model that can be coupled to/with a demand model, including no epidemiological model at all, we believe a direct comparison of predictions from different demand models would not be very informative, since any differences would likely be dominated by the epidemiological model and provide limited information on the demand calculator accuracy. Therefore, to provide a fair and more informative comparison, we decoupled the epidemiological aspect from the demand calculators. Thus, our analysis is split into two distinct stages.

First, we evaluated the predictive capability of a range of hospital use epidemiological models (that is, epidemiological models that predict either current hospitalizations or new admissions), since these are typically key components within demand models. For this analysis, we used a modified version of an epidemiological validation framework that we recently developed [22].

In the second stage of the analysis, we evaluated a range of demand calculators for selected medical resources. We decoupled the epidemiological aspect from the demand models and only ran the calculator aspect. We used ground truth hospitalization data as the calculator input, thus evaluating the performance of the calculators in isolation. We selected three medical resources for this analysis: ventilators, gowns (as one specific type of PPE), and diagnostic testing kits. For each of these resources, we assessed multiple published demand calculators, comparing the models to each other and to ground truth data when available.

Overall, our analysis quantifies magnitudes of errors that may occur for demand models and provides important contextual information on demand model limitations. Based on our analysis, we will conclude by proposing recommendations to advance the field of medical resource demand modeling.

## Methods

### Evaluation of hospital use epidemiological models

First, we conducted a detailed investigation of epidemiological COVID-19 models predicting hospital use, because these epidemiological models form the basis of predictive demand models. We did not restrict to the epidemiological models listed in Table 1, but rather examined hospital use epidemiological models in general. There are generally two types of hospital use models: (i) **hospitalization models**, which predict the number of patients currently hospitalized on a given day and (ii) **admission models**, which predict new admissions on a given day. Both current COVID-19 hospitalizations and COVID-19 hospital admissions are important measures as they account for various movements of patients in a hospital setting.

We used the framework of Dautel et al. [22] with some modifications to evaluate these hospital use models. Briefly, that framework is a novel validation framework for epidemiological models focused on accuracy of quantities that end-users may find important, such as date of peak, magnitude of peak, or time to recovery. Several validation metrics were proposed that quantify overall model accuracy of these quantities. The framework accounts for the fact that epidemiological models used by public health decision makers typically have regular releases of new predictions, rather than the model being a single set of predictions. For example, one metric is error in predicted date of peak, as a function of release date. In Dautel et al. [22] we applied the framework to evaluate the accuracy of four prominent COVID-19 death models and one COVID-19 hospitalization model, for the period of Spring and Summer 2020; these were the only models that provided the necessary data for this time period. Therefore, the results in [22] provide limited information about hospital use models and it was not possible to derive any links between modeling approach and accuracy. In addition, Dautel et al. [22] does not distinguish between a model overestimating versus underestimating; however the difference is critical for demand models.

Therefore, for this paper we applied a modified version of the validation framework to a wide range of hospital use models. The time period considered in this work is late 2020 to early 2021, the so-called Fall COVID-19 wave, when more models were predicting COVID-19 hospitalizations/admissions and publicly shared their past and current predictions. We refined the previously defined metrics to distinguish between underestimation and overestimation. Unlike Dautel et al. [22], we also examined how a model’s structure impacted its performance. The process we followed is summarized below.

#### Ground truth and models

Our ground truth data source of COVID-19 hospitalizations was from HealthData.gov where data is provided from the US Department of Health and Human Services [23]. We looked at only confirmed COVID-19 hospitalizations, not suspected.

The models we analyzed were based on which groups provided sufficient information for us to apply the validation framework. Sufficient information includes access to the raw model predictions for the latest release and all previous releases, which the framework requires. Relatively few modeling groups made previous predictions publicly available. Of the fifteen models listed on the CDC website [24] between the dates of September 2020 and April 2021 we identified two hospitalization models and eight admissions models satisfying these criteria; nine modeling groups in total. The models are listed in Table 2. Of these, two models were excluded as they did not provide sufficient predictions for the period analyzed. Overall, we analyzed two hospitalization models and six admissions models in total. Note that in this paper, we use ‘model’ to refer to predictive tools developed by organizations, not the underlying modeling approach. We have not implemented and do not run any particular model ourselves. Comparison of different modeling approaches such as performed in [25] is not the goal of the validation framework and out of the scope of this document.

**Table 2:** List of hospital use models evaluated

Name	Model output	Release period	Prediction period	Comments
Columbia University Shaman group (Columbia) [26]	Hospitalizations	April 2020 - March 2023	42 days	
Covid-19 Simulator Consortium (Covid19Sim) [27]	Admissions	May 2020 - March 2022	130 days	
Georgia Institute of Technology, College of Computing (GT-DeepCOVID) [28]	Admissions	May 2020 - June 2023	30 days	
Institute for Health Metrics and Evaluation (IHME) [29]	Hospitalizations and admissions	March 2020 - December 2022	130 days (hosp) and 103 days (admis)	
Johns Hopkins University Applied Physics Lab (JHU APL Bucky) [30]	Admissions	August 2020 - August 2022	60 days	Did not have admission predictions in releases after 12/21/20 for all peak events examined. Therefore, did not continue with analysis.
Johns Hopkins University Infectious Disease Dynamic Lab (JHU IDD) [31]	Admissions	April 2020 - ongoing	41 days	
Karlen Working Group (Karlen) [32]	Admissions	July 2020 - February 2023	30 days	
University of California, Los Angeles (UCLA) [33]	Admissions	May 2020 - June 2022	100 days	No admission predictions for Florida, Georgia, and Ohio peak events.
University of Southern California (USC-SI kJalpha) [34]	Admissions	June 2020 - February 2023	56 days	Admissions releases do not start until 11/22/20. Therefore, did not continue with analysis.

Epidemiological models typically provide a measurement of uncertainty in their forecasts, such as 90% confidence intervals or selected percentiles. All models in Table 2 provided probabilities for each prediction. However, the validation framework in [22] only evaluates the accuracy of point predictions and does not consider the uncertainty estimates. This is an acknowledged limitation in [22] and similarly a limitation of the present work that will be discussed further in the Discussion section. The model results analyzed in this paper were the median predictions in each release.

#### Selection of peak events

**Fig. 1:**
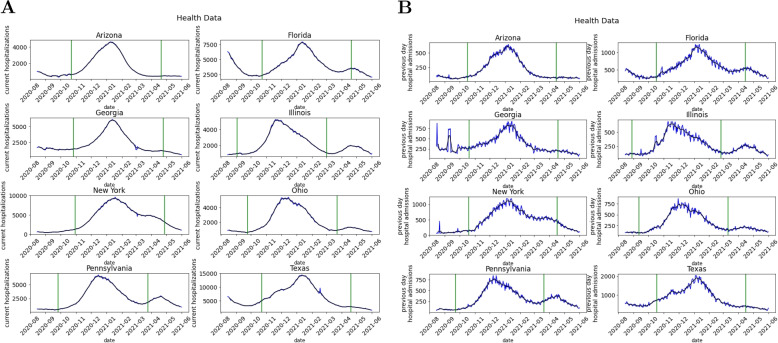
HealthData.gov information during Fall 2020 peak events for current hospitalizations (**A**) and admissions (**B**). Blue line represents daily data and black line represents 7 day rolling average. Left green line is 80 days before the peak and right green line is 100 days after the peak

The framework requires selecting local ‘peak events’, which are regions and time periods of increasing and then decreasing COVID-19 hospitalizations/admissions (i.e., COVID-19 ‘waves’). These were selected as follows. We considered the period of late 2020 to early 2021, henceforth referred to as Fall 2020. Hospitalization time series for each U.S. state were assessed for candidate events by eye, for periods with a clear rise and decrease in hospitalizations. Candidate events were retained as peak events if they satisfied the following: (i) maximum seven-day average was greater than 5000; (ii) seven-day average was less than 50% of maximum value in period prior to peak; (iii) seven-day average decreased to less than 50% of maximum value in period after peak. The start and end dates for each hospitalization peak event were chosen based off the hospitalization seven-day average peak date. The start date was set to be 80 days before the hospitalization peak and the end date was 100 days after the peak date. We utilized these same peak events with the same start and end dates for hospital admissions. Table 3 lists the identified peak events, which are plotted in Fig. 1.

**Table 3:** Peak events for currently hospitalized and daily hospital admissions

State	Start Date	End Date	Hospitalizations 7-day avg peak	Admissions 7-day avg peak
Arizona	10/23/2020	4/21/2021	1/11/2021	1/6/2021
Florida	10/23/2020	4/21/2021	1/11/2021	1/6/2021
Georgia	10/27/2020	4/25/2021	1/15/2021	1/9/2021
Illinois	9/3/2020	3/2/2021	11/22/2020	11/18/2020
New York	10/30/2020	4/28/2021	1/18/2021	1/8/2021
Ohio	9/24/2020	3/23/2021	12/13/2020	12/2/2020
Pennsylvania	9/26/2020	3/25/2021	12/15/2020	12/12/2020
Texas	10/22/2020	4/20/2021	1/10/2021	1/7/2021

#### Validation workflow

Our workflow is similar to our work in Dautel et al. [22] with some adjustments. First, a statistical spline-based model was fitted to each of the 16 ground truth peak event time series, using the Markov Chain Monte Carlo method as outlined in Dautel et al. [22]. From these we inferred confidence intervals for the true peak date and magnitude; see [22] for details. Unlike [22] we next qualitatively assessed model performance prior to computing the quantitative validation metrics. This is because for the hospital use models in this paper, many model releases did not predict any peak occurring, unlike the death models in Dautel et al. [22] which consistently predicted a peak and subsequent decrease. When no peak is predicted, validation metrics, based on the difference between predicted peak date and true peak date, are undefined. Therefore some models did not continue past the qualitative assessment. For models which passed through to the next stage, we quantified predictive accuracy of peak date and peak magnitude using the adjusted $\texttt {PeakDate\_Error}$ and $\texttt {PeakMagnitude\_Error}$ metrics in Dautel et al. [22]. $\texttt {PeakDate\_Error}$ refers to the error in the predicted peak date as a function of model release date and $\texttt {PeakMagnitude\_Error}$ refers to error in the predicted peak magnitude as a function of release date. We adjusted the metrics to distinguish between under and over-estimation by replacing the area metric in Dautel et al. [22] with a signed area metric. Finally, we examined forecasts for total hospitalizations and admissions; specifically, totals hospitalizations/admissions over the 28-day period following a model release, termed ‘future cumulative hospitalizations/admissions’. Future cumulative hospitalizations is an important quantity for a model to predict because it determines the quantity of select medical resources needed as various healthcare workers interact with these patients throughout their time in the hospital. Future cumulative admissions is important to ensure there is hospital bed availability and an adequate amount of scheduled staff to care for incoming patients.

### Demand calculator analysis approach

The demand calculator analysis is completely distinct from the epidemiological model analysis and involved considering selected models from Table 1 and de-coupling the calculators from their underlying epidemiological models. We provided true values to the demand calculator as inputs (i.e., true hospitalizations or ICU bed use), and then assessed the calculator performance, either by comparing predictions against data on actual resource use, or, when such data were unavailable, comparing calculators against each other. This assessed the calculators under the best case of an ideal epidemiological model. We focused the analysis around three important resources: ventilators, gowns and diagnostic tests, considered below in turn.

#### Ventilator demand models

We first considered the accuracy of demand calculators in ventilator demand models. There are three demand models listed in Table 1 that provide ventilator predictions for the US. These three models have a similar approach to predicting the number of patients requiring ventilators by scaling either number of hospitalizations or number of patients in the ICU. Therefore, these ventilator demand calculators are simply a single parameter scaling of the epidemiological model output.

Therefore, for ventilator models, the calculator accuracy will be dependent on the assumption of a constant fixed parameter and on the accuracy of this parameter. To investigate this, we utilized publicly available data on the number of patients with COVID-19 currently in the ICU and the number of patients with COVID-19 on a ventilator and compared this ratio against the value used in the demand calculators. Since these are simple one-parameter models, this is equivalent to passing the true ICU usage into the calculator and comparing predicted ventilators with true ventilator use, which is the approach that will be used for gowns and diagnostic tests. Covid Tracking Project’s data [35] on current patients with COVID-19 on a ventilator was used to obtain the ratio for each state during the given time period. If a state did not report data on either current hospitalizations or current ventilator usage, then the state was not included in the analysis.

#### Gown demand models

Next, we considered models predicting demand of medical gowns. In Table 1 there are ten models that predict gown usage. Of these, six were not analyzed due to either lack of data or opacity of underlying methods. The four gown demand models analyzed were the WHO, Penn, JHU and CNH models. For the CNH model we examined two model variants based around different conservation strategies of gowns: liberal and moderate. Liberal use is where no PPE is re-used. Moderate conservation is where each healthcare worker reuses each article across every interaction with the same patient.

Due to a lack of data on actual gown usage we focused on comparing the four models to see how much their predictions varied. However, the regions of interest, inputs and assumptions are different between the four demand calculators. Therefore, we had to ‘align’ the calculators to enable a direct comparison, which included scaling up the smaller-scale models to take in state/nation level inputs to predict state/nation level outputs, which assumes the smaller-scale models assumptions apply to the larger scale.

First, we compared the WHO and JHU models which are designed to predict on the scale of the US as a whole. We analyzed their predictions during the Fall 2020 COVID-19 peak, and utilized data from the CDC [36] for calculator inputs. The CDC data set reported both COVID-19 cases and deaths daily for each US state as well as the entire US. The WHO model required cumulative weekly cases as input which we provided for each week in the period of interest. The JHU model required deaths as input. Weekly deaths were provided to the JHU model, rather than the entire wave as intended in the tool, to enable a direct comparison with the WHO model. Additionally, to make a fair comparison, nursing homes and EMS gown usage were removed from the JHU model predictions.

Second, we compared all four models for Fall 2020 for New York state (NY) and the US as a whole. This again required alignment of the models as they differed in scope (e.g., one hospital versus entire US healthcare system) and temporal scale (e.g., daily versus weekly).

For the WHO model, weekly cumulative case counts for NY and the US were needed as input to predict the number of gowns needed, using data from the CDC [36]. The output was weekly gown demand for NY and the US, which was converted to average daily number of gowns needed for a given week. The same process was used for the JHU model, except as above, nursing home and EMS gown demand were removed. The CNH and Penn models provide daily predictions and require inputs related to currently hospitalized patients at the beginning of the day and admitted over the course of the day for one hospital. To scale predictions up to the state and country level, we provided inputs corresponding to all hospitals in NY and the US, using data from the New York State Department of Health [37] for NY and from the CDC [38, 39] for the US. Seven day averages of daily current COVID-19 hospitalizations and daily newly reported COVID-19 hospital admissions quantities were provided as input into the CNH model (just hospitalizations) and the Penn model (hospitalizations and admissions). Additionally, the Penn model required input of number of COVID-19 patients in the ICU, for which data from [37] and Our World in Data [40] was used. Thus, the CNH and Penn models were made to predict average daily gowns needed each week for NY and the US, and could be compared to the WHO and JHU models.

#### Diagnostic testing kit demand models

The final resource we investigated was diagnostic tests. There is one model in Table 1 that predicts COVID-19 diagnostic test usage, the WHO model. This model predicts the total number of polymerase chain reaction (PCR) and antigen testing kits used, and is dependent on weekly number of COVID-19 cases. The WHO model has two different testing strategies: targeted and all suspected cases. A targeted strategy is where tests are only provided for the proportion of cases classified as severe and critical plus an additional user-defined percentage (we used the default value of 10%) of moderate and mild cases to allow testing of vulnerable populations at-risk. An all suspected cases testing strategy assumes that tests will be administered to all presenting suspected cases. While we did not identify any dataset providing actual tests used, data was available on shipped molecular testing kits over the period from April 2020 to October 2022 [41]. This data set was collected by the Advanced Medical Technology Association (AdvaMed) and accounts for thirteen leading diagnostic manufacturers whose tests together comprise approximately 75–80% of the COVID-19 molecular in vitro diagnostic tests on the market in the US. This data was utilized as a surrogate for tests used. We used the WHO model to predict tests needed on a weekly basis over the same time period, using the same approach as the  Gown demand models section, and compared the model predictions with the shipped tests data. Since this is not a like-for-like comparison, care is needed when interpreting the results.

## Results

### Evaluation of hospital use epidemiological models

First, we present the results of the hospital use epidemiological models. Table 4 summarizes the results presented in this section and also includes each model’s underlying modeling approach. The main goals of this section are to understand the general performance of the models and to compare their results; it is in this context that we frame the below discussion.

**Table 4:** Summary of each hospital use model’s performance

	Model	Approach	Predicted a peak	Peak date estimation	Peak magnitude estimation
Hospitalizations	Columbia	Metapopulation SEIR model	Yes	Initially under, later over	Initially under, later over
	IHME	Co-variate driven SEIR combined with spline	Yes	Over	Over
Admissions	Covid19Sim	SEIR model	Yes	Over	Initially under, later over
	GT-DeepCOVID	Deep learning	No, generally monotonically increasing	N/A	N/A
	IHME	Co-variate driven SEIR combined with spline	Yes	Over	Under
	JHU IDD	Metapopulation SEIR model	Yes	Mostly under	Under
	Karlen	Discrete time difference equation	No, generally monotonically increasing	N/A	N/A
	UCLA	Modified SEIR model	Yes (but often flat or quickly decreasing from start date)	Mostly under	Under

#### Qualitative analysis

**Fig. 2:**
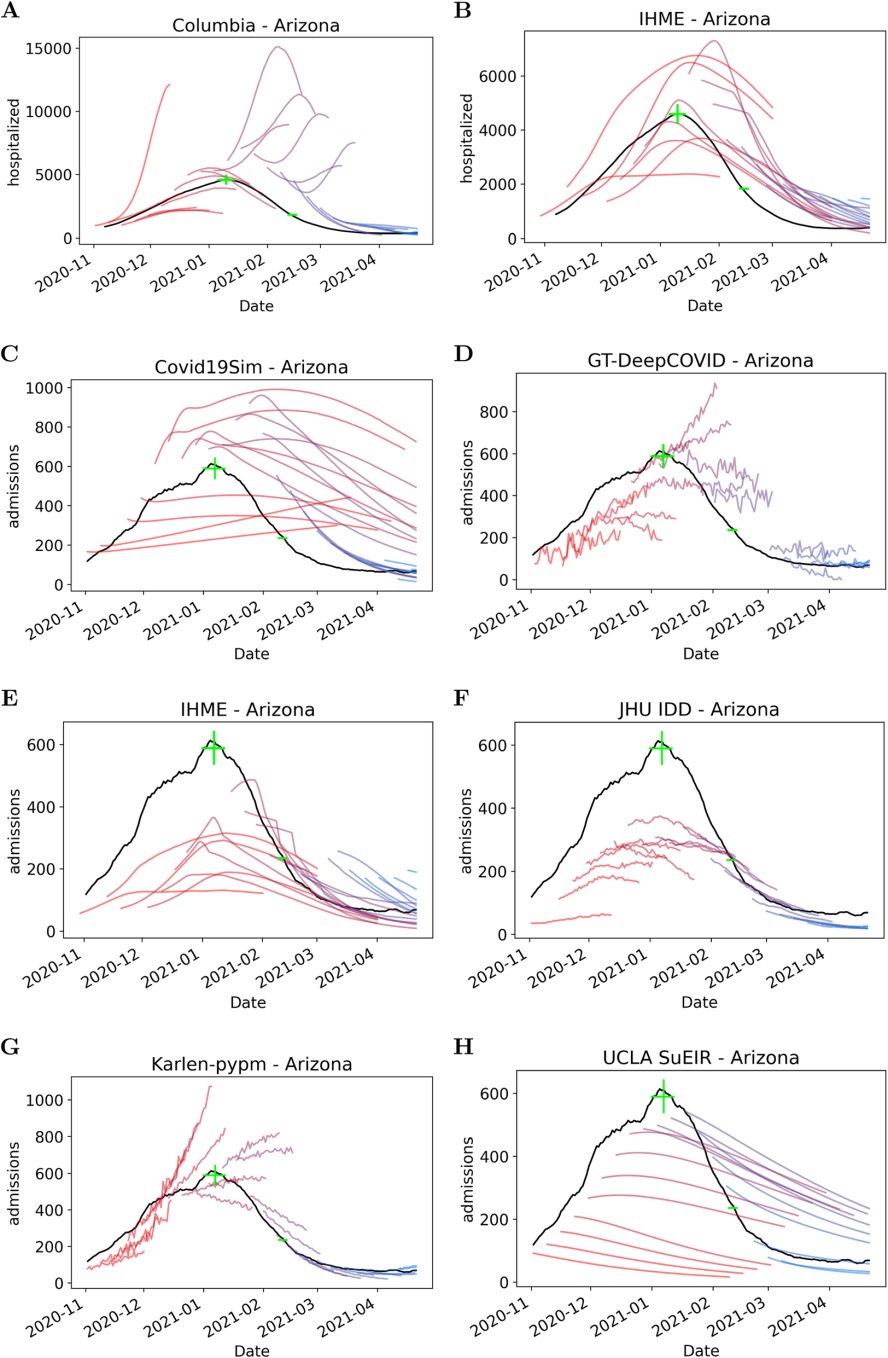
Predictions for Arizona during Fall 2020 for each model. Black line is the seven-day rolling average for recorded number of hospitalizations/admissions and green lines represent uncertainty in true peak date and magnitude. Red lines indicate early model releases; blue lines indicate later model releases; purple lines are intermediate results

Figure 2 provides sample results of the epidemiological qualitative analysis. Figures were generated for each peak event and each model, but only the Arizona results are presented, as a representative example. Results for the other peak events are provided in Additional File 1. Below, we summarize the general trends observed from each model. While these trends are apparent in the representative figure (Fig. 2), they are based upon the plots for each of the eight peak events for each model. Note that the UCLA model did not have admission predictions for all peak events. Peak events excluded from analysis for this model include Florida, Georgia, and Ohio. Conclusions for the UCLA model are based on the remaining five peak events.

**Hospitalization models**: Predictions from the two hospitalization models are not particularly similar. Columbia’s (Fig. 2A) early model releases (indicated in red) often did not predict far enough out to infer a peak. Later releases occurring around and after the true peak occurred often had significantly overestimated peak magnitude. In general, the IHME model (Fig. 2B) more closely matched the ground truth. It usually predicted far enough out to infer a clear peak, and while there was a mixture of underestimation and overestimation of the magnitude of hospitalizations, it followed the general trend of the ground truth data.

**Admissions models**: Fig. 2C-H illustrate how the admissions models predictions are very different and can fail to match the ground truth but in different ways. For example, some models predicted essentially linear trends for a given release (GT-DeepCOVID and Karlen, Fig. 2D and G) meaning they did not predict any peak occurring. The Karlen model did however capture the dynamics at the end of the time period analyzed. The IHME and JHU IDD models (Fig. 2E and F) were similar for Arizona, both predicting peaking but underestimating peak magnitude. In general, the JHU IDD model predictions were highly dependent on the peak event (see Additional file 1). It rarely overestimated and often performed well at capturing the timing of the rise and fall of the peak.

Regarding the remaining two admissions models, early releases of the Covid19Sim model (Fig. 2C) had predictions that linearly increased with a relatively flat slope. This model’s predictions rarely followed the trend of admissions truly occurring. Almost all releases of the UCLA model (Fig. 2H) that were made before the true peak had occurred were a monotonically decreasing trend. Therefore the magnitude of admissions was almost always underestimated.

Overall, it is evident from the variability across Fig. 2C-H (and corresponding results for other states, see Additional file 1) that demand model predictions could vary enormously based on the underlying epidemiological model.

The next stage of the assessment quantitatively compares predicted peak date/magnitude with observed peak date/magnitude. However, as seen in Fig. 2, the GT-DeepCOVID and Karlen models often did not predict any peak date. Therefore, these two models did not continue to the next stage of the analysis.

#### Accuracy of peak date and peak magnitude

**Fig. 3:**
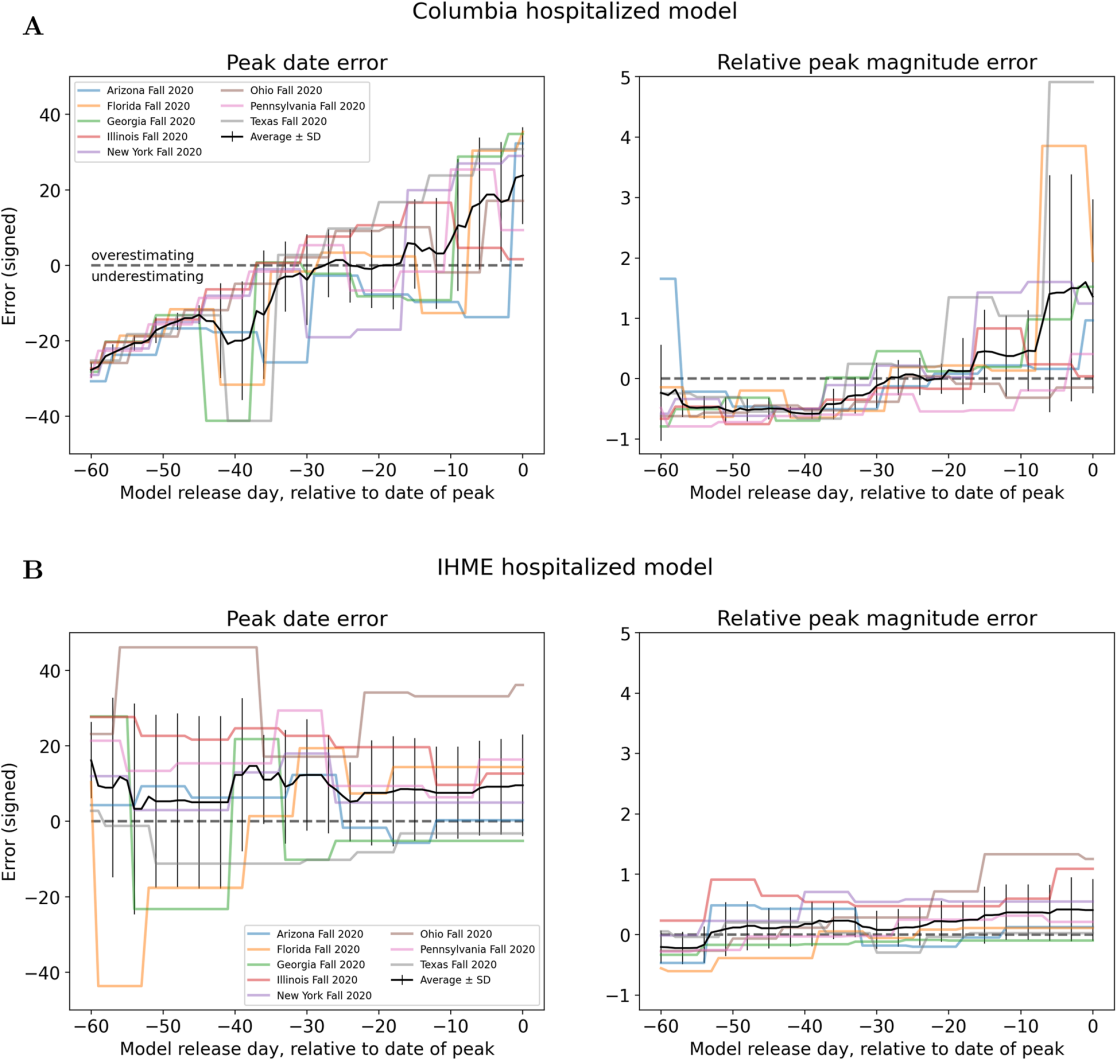
Hospitalization model errors in peak date and peak magnitude for (**A**) Columbia and (**B**) IHME. Colored lines represent different peak events, black line is average across peak events

**Fig. 4:**
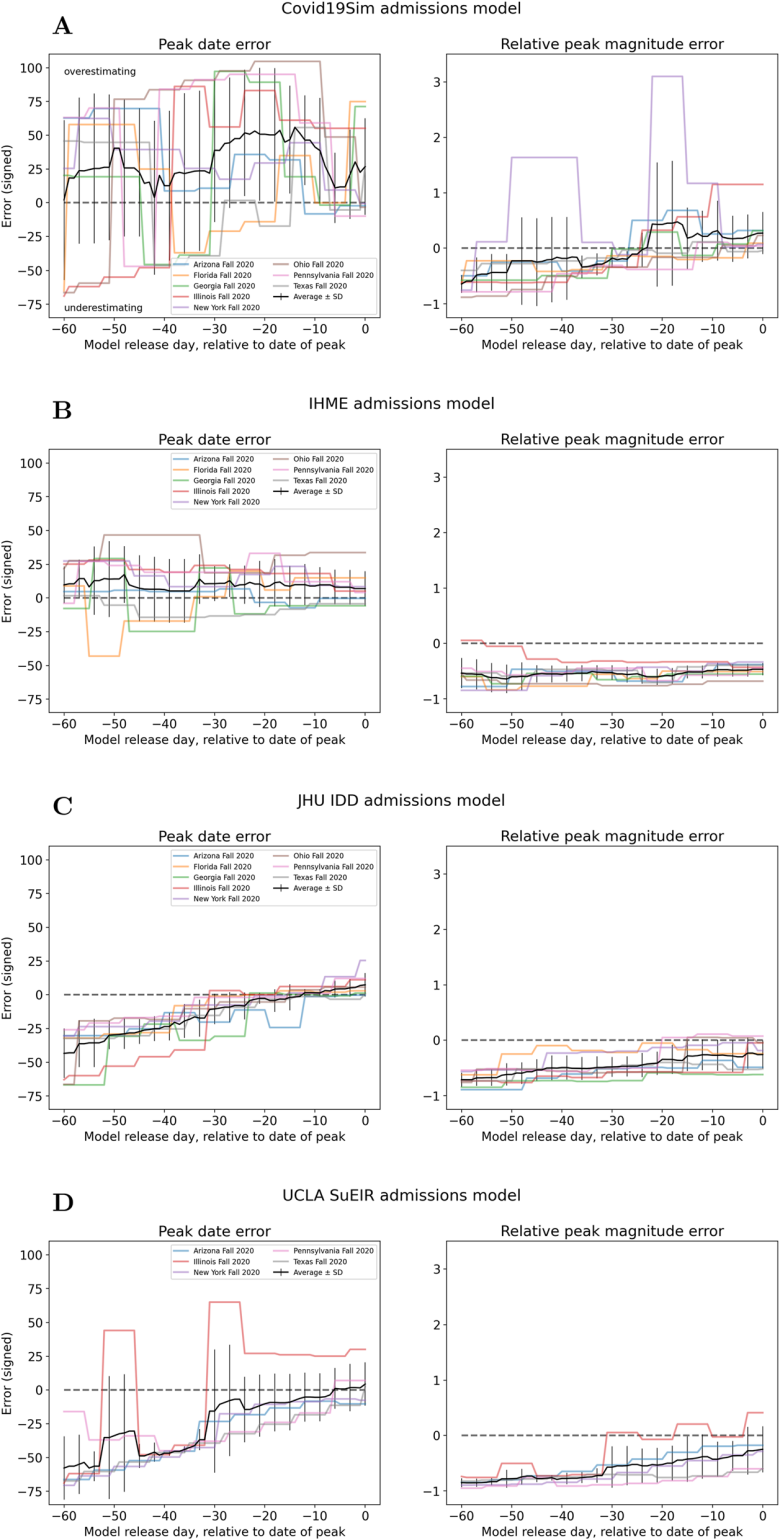
Admission model errors in peak date and peak magnitude for (**A**) Covid19Sim, (**B**) IHME, (**C**) JHU IDD, and (**D**) UCLA. Colored lines represent different peak events, black line is average across peak events

**Fig. 5:**
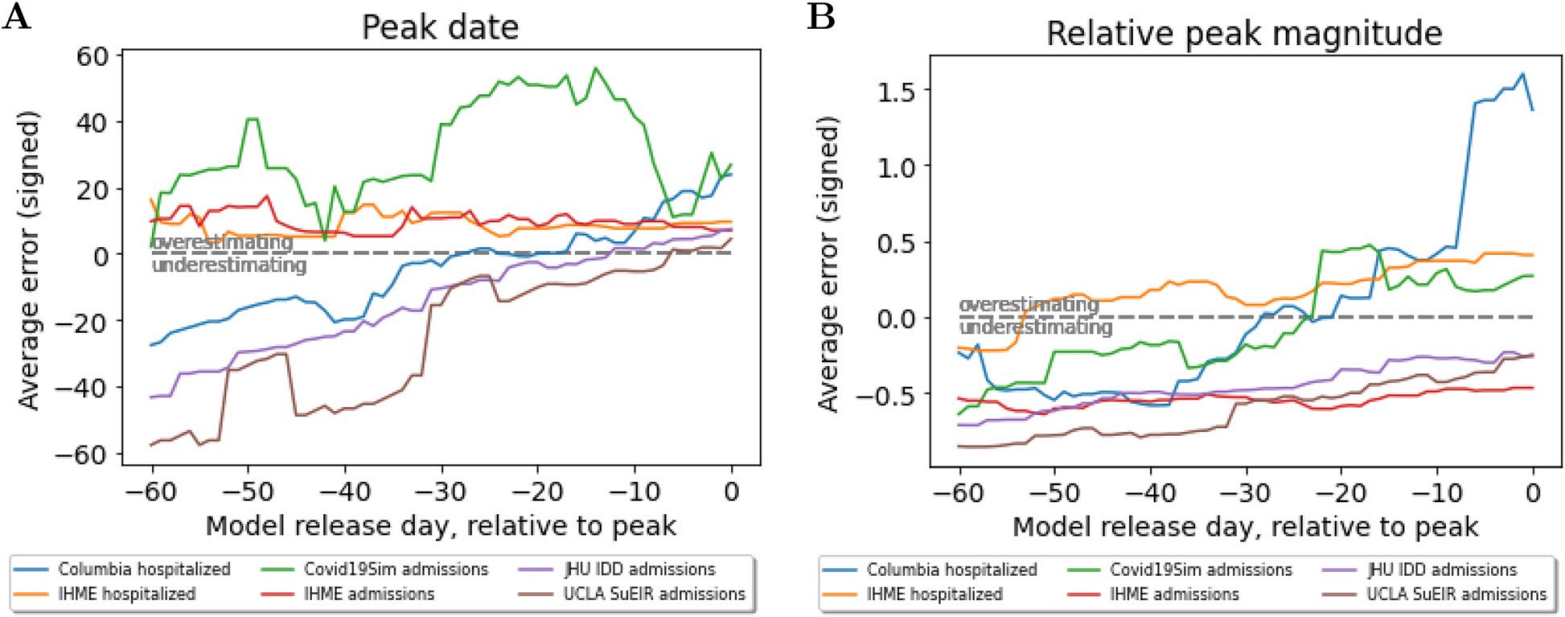
Average signed error of (**A**) peak date and (**B**) relative peak magnitude for all hospital use models

Next, the signed error in predicted peak date and peak magnitude is computed for each model and each peak event, plotted in Figs. 3 and 4. Only predictions released before the true peak are considered. Figure 5 provides a comparison of the average errors of the six hospital use models analyzed. Note that average errors (black lines in Figs. 3, 4 and 5) need to be interpreted with care when near zero, as this could imply either low errors across all peak events or underestimation for some peak events cancelling out with overestimation for others (this applies to the IHME results in Fig. 5A).

**Hospitalization models**: Fig. 3A shows that Columbia’s error for peak date increased almost linearly, with underestimation of peak date for earlier releases and overestimation for later releases. The signed peak magnitude relative error was enormous for late releases that occurred one week before the true peak date occurred. In contrast, IHME (Fig. 3B) predictions were very variable across peak events, on average overestimating peak date and not decreasing in magnitude nearer true peak date. The same comments apply for IHME peak magnitude predictions.

**Admissions models**: Fig. 4 plots the average error in peak date and peak magnitude for each of the four remaining admission models. For peak date, the Covid19Sim and IHME models have similar behavior (large variability across peak events, on average overestimating) and the JHU IDD and UCLA models have similar behavior (mostly underestimating, error linearly decreasing). All models generally underestimated peak magnitude, except Covid19Sim from about 20 days before the true peak date.

#### Accuracy of future cumulative hospitalizations/admissions predictions

**Fig. 6:**
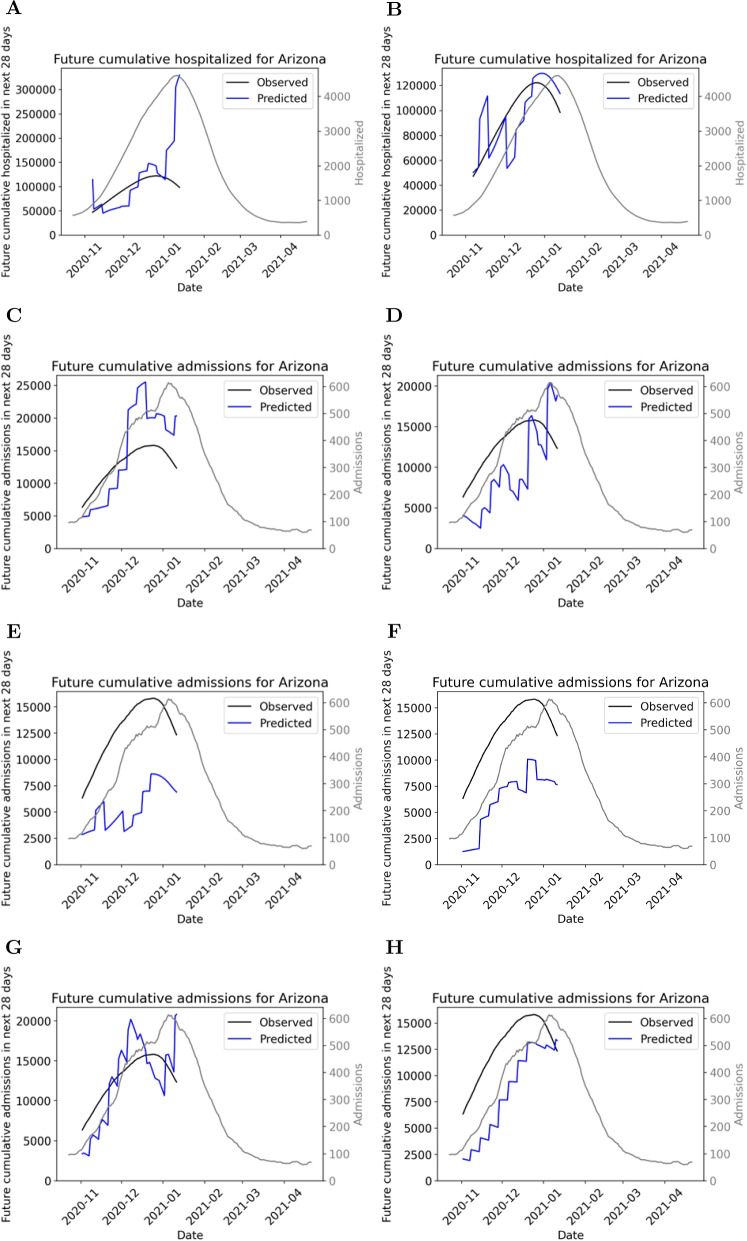
The future cumulative hospitalization predictions over a 28-day period following a model release for (**A**) Columbia and (**B**) IHME; and future cumulative admission predictions over a 28-day period following a model release for (**C**) Covid19Sim , (**D**) GT-DeepCOVID, (**E**) IHME, (**F**) JHU IDD, (**G**) Karlen, and (**H**) UCLA, all for the Arizona peak event. Blue line is model’s predictions, black line is true cumulative hospitalizations/admissions data from HealthData.gov. Also shown for context is daily hospitalizations/admissions (grey line)

Next, Fig. 6 provides the future cumulative hospital/admission predictions for Arizona, for each of the eight models, as a representative example. Results for the other peak events are provided in Additional File 1. Table 5 lists the error for each model, averaged over the dates considered and all peak events. As in the previous section, the below comments are based on the results for all peak events, not just Arizona.

**Table 5:** Values of normalized average future cumulative error

	Model	Average future cumulative error
Hospitalizations	Columbia	0.42 ± 0.12 (*n*=8)
	IHME	0.24 ± 0.08 (*n*=8)
Admissions	Covid19Sim	0.29 ± 0.12 (*n*=8)
	GT-DeepCOVID	0.49 ± 0.11 (*n*=8)
	IHME	0.59 ± 0.09 (*n*=8)
	JHU IDD	0.35 ± 0.17 (*n*=8)
	Karlen	0.39 ± 0.15 (*n*=8)
	UCLA	0.52 ± 0.14 (*n*=5)

Mean ± SD are presented, where the mean is average of metric across the peak events. n = number of peak events

Curiously, for the Columbia model, there was a drastic over-prediction near the end of the time period considered for seven out of the eight peak events (example in Fig. 6A). IHME’s future cumulative hospitalization predictions were generally fairly accurate to what was observed, hence a low average error. The performance of admissions models varied amongst each other. Covid19Sim’s predictions was generally fairly accurate to what was observed. All other models under-estimated future cumulative admissions either some or all of the time. For example, IHME drastically under-predicted future cumulative admissions for every peak event (example in Fig. 6E), hence the large errors in Table 5. JHU IDD under-predicted future cumulative admissions for Arizona, Georgia, Illinois and Texas; however, was quite accurate for other peak events. Karlen under-predicted for four out of five peak events. Finally, considering Table 5, the average errors lie in the 20-60% range. The lower error was from the IHME hospitalization model, which generally matched the true trajectory well and the highest error was from the IHME admissions model due to significant underestimation.

### Demand calculator analysis

#### Ventilators

**Fig. 7:**
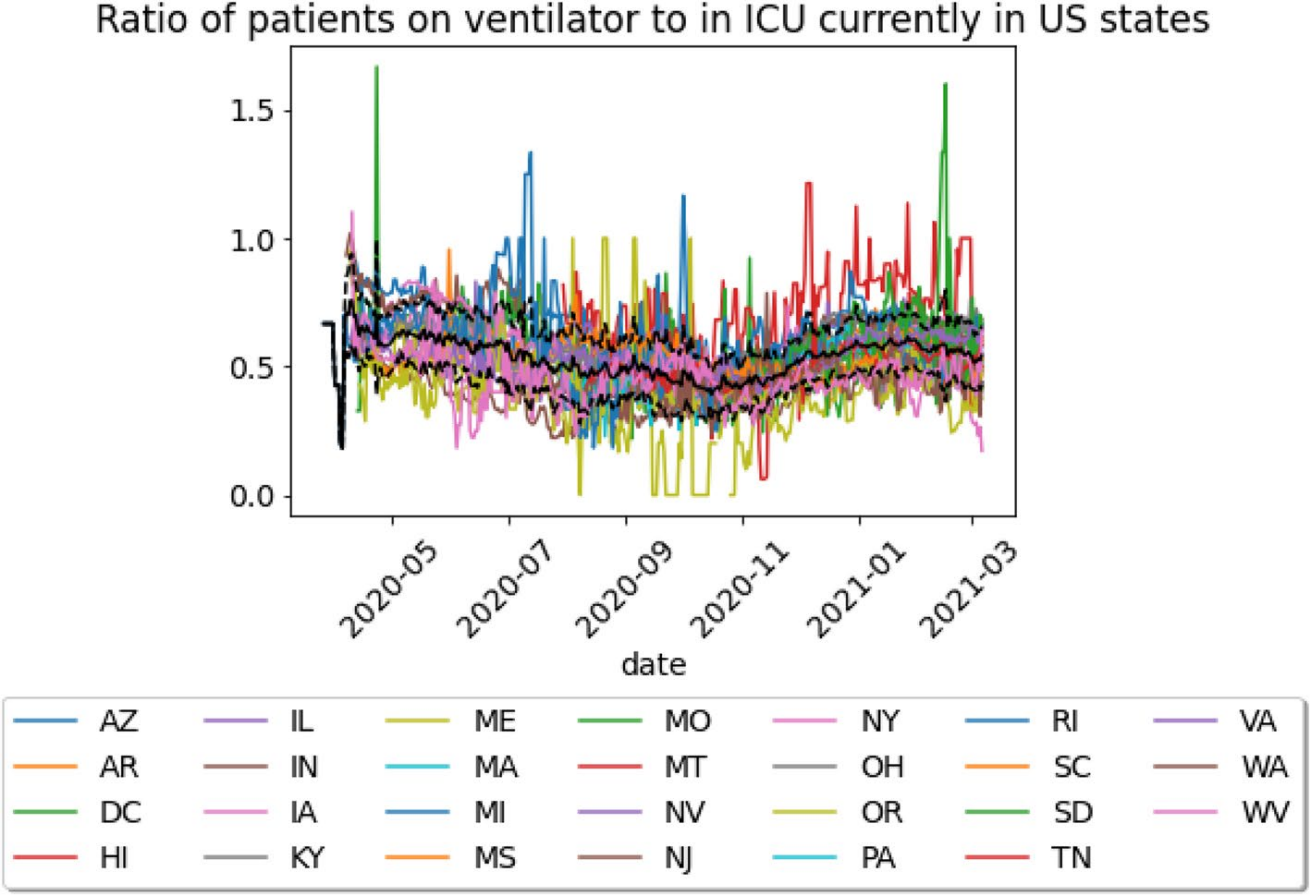
Ratio of number of patients on ventilator to number of patients in the ICU. Various colored lines represent ratio for different states from April 2020 to March 2021. Bold black line represents the average ratio across states that provided ventilator usage data. Dashed black lines represents 95% confidence interval

As stated in the Ventilator demand models section, three models in Table 1 predict ventilator demand. Wells et al. [7] developed a model in the early stages of the pandemic to illustrate potential ventilator use scenarios that might arise. In their model, they scaled ICU beds predicted by their epidemiological model by 0.544. A paper published by the IHME group predicted ventilator use by scaling ICU beds from the IHME epidemiological model by 0.54 [9]. An epidemiological model developed by Penn Medicine, CHIME, allows users to specify the number of current COVID-19 hospitalizations in a region, the hospitalization rate, the regional population, and the hospital market share. Default values correspond to 0.66 percent of patients in the ICU are on a ventilator. These values can be compared to the observed ratio of patients on ventilators to COVID-19 patients in the ICU, plotted in Fig. 7 (note: the same data also plotted in Additional File 1 using multiple figures so individual traces can be discerned). It is evident that on average, the ratio is between the range of 0.4 and 0.75 depending on the time. A ratio that is greater than one, which is demonstrated by select states, indicates that there are additional hospitalized patients not in the ICU that are on a ventilator. All models used parameter values within that range. However, it is crucial to recognize that this value varied depending on the time period and the states reporting, whereas it was assumed to remain constant in the models. While the parameter values used in the models may be reasonable average values, the true value for a given hospital on a given day varies across quite a wide range, and there is potential for significant underestimation of ventilator need. This could be addressed by accounting for the uncertainty in the parameter in the predictions and outputting a ventilator usage distribution. If so, the prediction for ventilator usage would be a range rather than a constant value.

#### Gowns

**Fig. 8:**
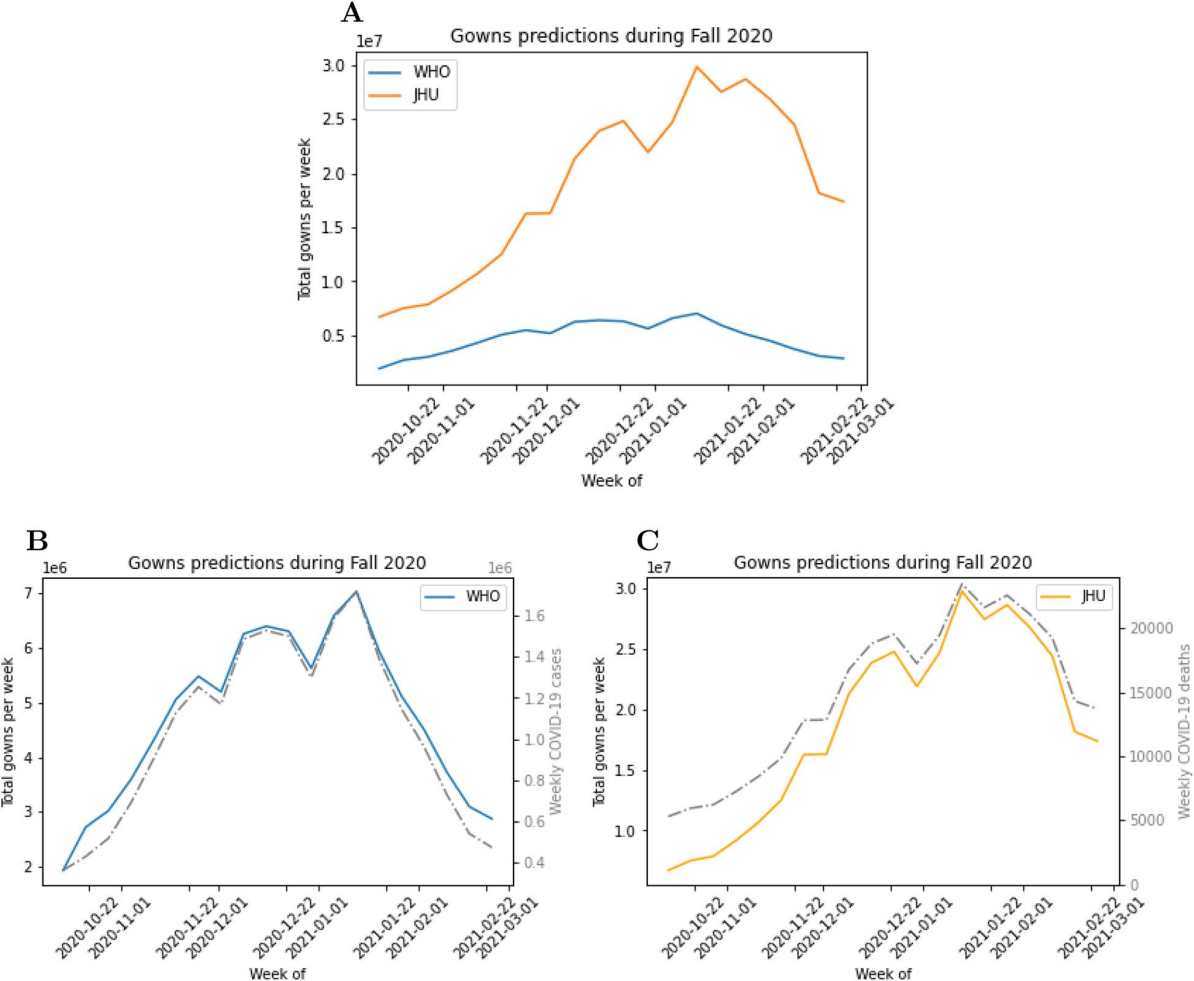
**A** WHO and JHU model gown estimates for US from October 14, 2020 through February 24, 2021, (**B**) WHO model gown estimates and its input of weekly COVID-19 case data, and (**C**) JHU model gown estimates and its input of weekly COVID-19 death data

The comparison of the WHO and JHU models, as well as their inputs, are displayed in Fig. 8. The gown estimates for the WHO and JHU models differ tremendously. At its maximum prediction of gowns needed in a week for the entire US, the WHO model predicts approximately 7 million gowns. In contrast, at its maximum the JHU model predicts 3 billion gowns needed in a week for the entire US. That is a substantial difference. The latter prediction corresponds to on average over 428 million gowns would be used throughout the US each day of that week. There are 6, 129 hospitals throughout the US as of 2022 [42]. This means there would be on average approximately 70, 000 gowns used each day on average during that week at each hospital. This would be an excessively large number of gowns used throughout the week. This large discrepancy is largely due to the vast difference in type of initial inputs these demand models use. JHU’s model scales the number of weekly COVID-19 deaths to obtain its output of gowns needed that week. The JHU model equates the number of COVID-19 ICU admissions as 14% of deaths, COVID-19 hospital admissions as four times the number of COVID-19 ICU admissions, and COVID-19 cases as 6.67 times the number of COVID-19 hospital admissions. The WHO model however, takes in cumulative weekly cases as inputs which then gets scaled to the number of hospitalizations and admissions. It is unclear which input is a better determinate of number of hospitalizations and admissions which would then relate to healthcare worker and patient interaction in which a gown would be necessary. Since there is no publicly available data on gown usage within hospitals during this time period of the pandemic it is not possible to quantify the errors in these predictions. The current inability to validate along with the wide range of model predictions raises concerns about the reliability of these predictions, discussed further in the Discussion section.

**Fig. 9:**
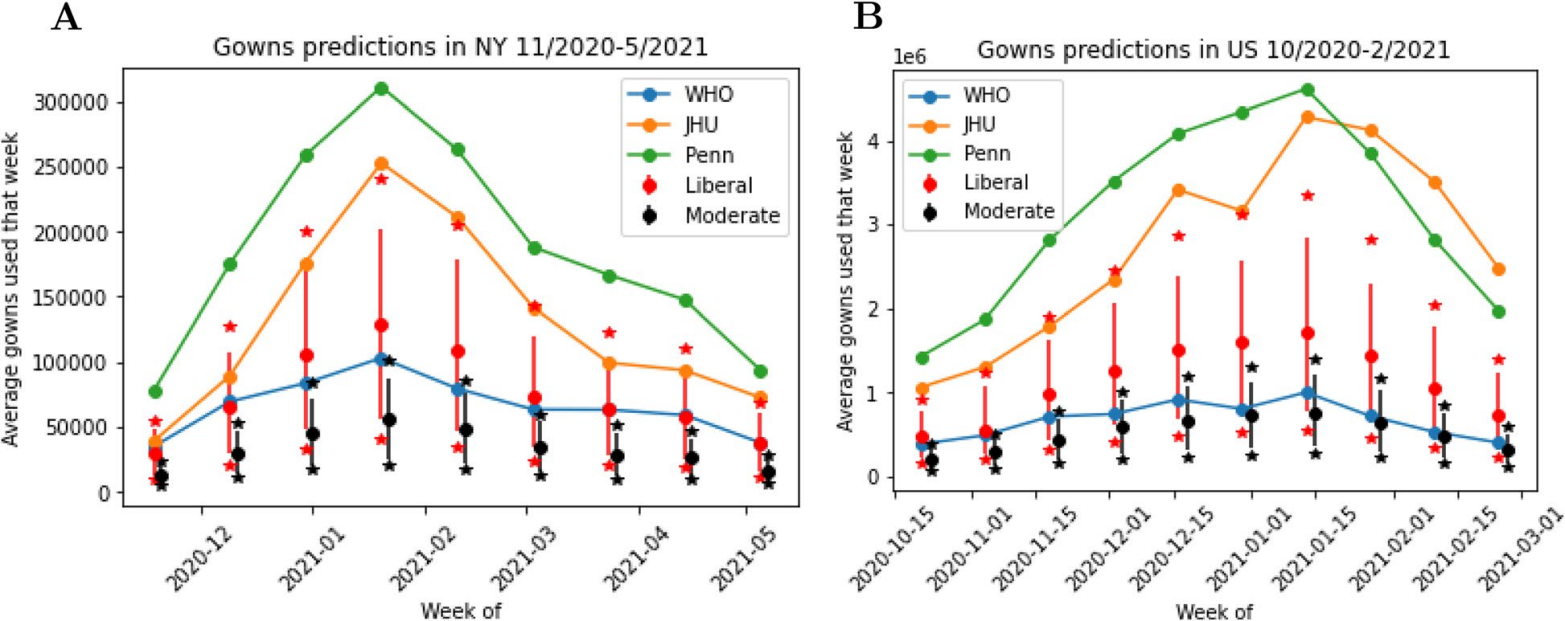
Gown demand model prediction comparison for (**A**) New York from November 2020 through May 2021 and (**B**) US from October 2020 through March 2021. The WHO, JHU, and Penn models are deterministic whereas the CNH model is stochastic with two variants, liberal and moderate conservation strategies. For the CNH model, the circle and bar represent mean and 90% confidence interval, and the stars represent the theoretical minima and maxima given the bounds of the uniform distributions for the model inputs

Figure 9 presents the comparison of the WHO, JHU, Penn and CNH models (two variants for the latter). Again, significant differences between the models are observed. The moderate conservation strategy for the CNH model had the lowest gown predictions, largely due to this model’s conservation strategy which allowed for the reuse of gowns by a healthcare worker when seeing a patient more than once. The Penn model had the largest gown predictions for NY and most of US during the time period analyzed. This is likely due to the breakdown of hospitalizations that are required as input into the demand calculator, which captures admissions, hospitalizations, and patients in the ICU. This calculator does not infer hospitalizations derived from other key quantities such as cases or deaths. The range of predictions amongst the four models for gowns demonstrates that the input of each demand calculator directly impacts the magnitude of gowns predicted.

#### Diagnostic tests

**Fig. 10:**
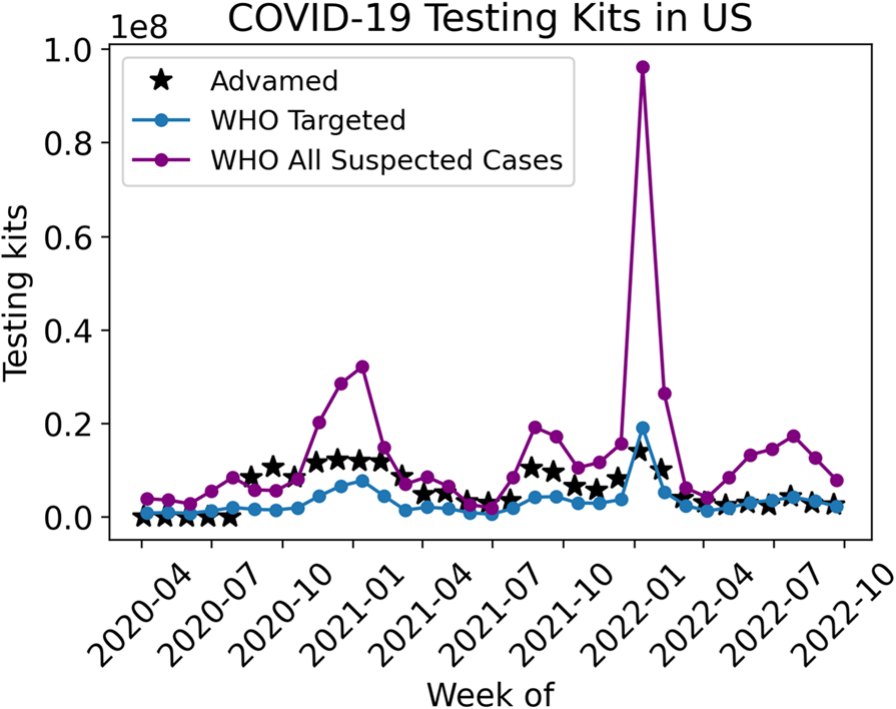
A comparison of WHO’s model predictions of two different testing strategies for diagnostic testing kits needed in the US and AdvaMed’s shipped molecular testing kits from April 2020 through September 2022

The comparison between WHO model diagnostic test demand predictions and AdvaMed shipping data is provided in Fig. 10. This is not a like-for-like comparison, since for example a wave of shipped COVID-19 diagnostic testing kits may not align with a wave of COVID-19 diagnostic testing kits being utilized. Moreover, the AdvaMed dataset accounts for molecular diagnostic tests, including polymerase chain reaction (PCR) and other nucleic acid amplification tests (NAATs), but the WHO model predicts for molecular testing kits and antigen tests. Note that rapid in-home antigen tests were not widely available in the early months of the time window considered, but were in wide-scale use in 2022. Furthermore, AdvaMed does not account for 20–25% of the manufactured and shipped COVID-19 molecular tests.

Therefore, we should not expect the data and predictions to align exactly. It is notable though that for the majority of the time period analyzed the predictions are similar in magnitude to the data, and reported data lies between the two model predictions. However in January 2022, there is a large discrepancy between COVID-19 molecular tests shipped and predicted diagnostic testing kits. The predicted number of suspected cases by the WHO model substantially increased causing a need for individuals to test. This prediction is based without a cap on the availability of a testing kit. However, the AdvaMed data does not show as drastic a spike in shipped diagnostic tests. During this time the discrepancy could be explained by individuals utilizing a diagnostic kit previously obtained. Additionally, during this time individuals could be continuously testing as they may have been in contact with multiple different individuals who have contracted COVID-19.

## Discussion

In this paper we analyzed the reliability of multiple COVID-19 medical resource demand models. To our knowledge this is the first analysis of its kind. In this section we discuss our findings and provide recommendations for advancing the field of medical resource demand modeling.

First, we analyzed eight different hospital use models utilizing the validation framework developed in Dautel et al. [22]. Performance varied greatly across models. We observed more variability in performance of hospital use models than death models in Dautel et al. [22]. In addition, there were some poorly performing models that did not predict any peak (see Fig. 2D and F). These types of discrepancies between predicted and actual hospital demand could lead to substantial errors in resource demand predictions. An under-prediction in admissions could lead to healthcare workers being unequipped to work with the number of people entering the hospitals each day. An over-prediction in admissions would likely lead to hospitals being well prepared but could potentially take away medical resources from other hospitals in need.

Surprisingly, admissions models did not perform better than hospitalization models, despite being simpler in principle. Overall, the typical error in predicting peak date, across hospital use models, was 2-4 weeks for earlier model releases, in either direction (i.e., no general under or over-estimation). The typical error in predicting peak magnitude (for earlier model releases) was in the 50% range. The peak magnitude was often underestimated by the models. Therefore, one could expect (current) demand models to underestimate true resource demand by 50% or more, and miss the period of greatest demand by up to a month.

Table 4 compared the performance of the hospital use models with their underlying modeling approach. IHME’s and Columbia’s hospitalization models are both modified SEIR models. However, IHME performed better at predicting future cumulative hospitalizations than Columbia’s hospitalization model (Table 5), and in general peak date and magnitude errors were smaller for IHME than Columbia. This could be due to the foundation of IHME’s model which is grounded in real-time data and is updated frequently. When examining Columbia’s model the predictions that incorporated no contact reduction estimates were analyzed which may have affected the model’s overall performance. Covid19Sim performed better at predicting admissions compared to other models analyzed, as seen in Fig. 5. Covid19Sim is also an SEIR compartmental model. This model is different from others of similar structure in that it is calibrated to reproduce historic trends in daily reported cases and deaths, and is updated weekly as new data and evidence arise. This likely aided in its leading performance. Compartmental model performance varied which demonstrates that parameter values, calibration to historic trends, and spatial components are important attributes that can enhance or diminish how well a compartmental model performs. Karlen and GT-DeepCOVID were two non-compartmental models examined, and used machine learning approaches. However, these models did not predict any peak in many cases and did not continue past the qualitative analysis in the Qualitative analysis section.

We also evaluated the predictions of the demand calculator aspects of demand models, for a selection of resources. We decoupled the demand calculators from underlying models and provided ground truth data as inputs to evaluate the calculators under a theoretical best-case scenario. For gowns, for which there was no comparator validation data on actual gown usage, we observed large differences between the models. For ventilators we observed that the simplicity of the models may not adequately capture real world variability and providing uncertainty in predictions could be important. For diagnostic tests, we compared model predictions with a shipped tests, as a surrogate to tests used, and observed similar trends on the two. Overall, our results support the utility of demand models but it is clear that large errors are possible, perhaps of the order of 100%, due to a combination of errors arising from both the epidemiological model and the demand calculator.

This paper has focused on demand modeling in the context of emergency situations such as epidemics or pandemics. However, in principle demand calculators could be useful in supporting resource allocation under normal hospital operating conditions, provided a suitable seasonal respiratory virus model was available to provide inputs to the demand calculator. We expect the approaches to model evaluation presented in this paper to be fully applicable to such cases.

The epidemiological models we assessed provided time series predictions. Some efforts have begun to focus on decision-relevant quantities directly. For example, in the FluSight forecast hub run by the US CDC [43], the models report probabilities that weekly flu hospitalization rate will have a large increase, moderate increase, large decrease, moderate decrease, or remain stable. The validation framework we used could be extended using additional metrics, to validate such predictions as a function of release date.

One limitation of this work is that the uncertainty ranges provided by the epidemiological models were not accounted for in the hospitalization/admissions model evaluation. This is because the validation framework we used, developed in [22], only considered the accuracy of the model point predictions. A future improved version of that framework that includes model uncertainty would provide more nuanced information about the models’ predictive capability. Alternatively, traditional model performance scores that account for uncertainty, such as the weighted interval score (WIS), could be used in concert with the validation metrics of [22] for a holistic view of the model. Note that the model validation paradigms of [22] and WIS are quite different, since the latter considers all model releases at once whereas WIS has to be applied to a single release. For demand calculators, none of the tools we analyzed provided uncertainty estimates, except the CHN model. In fact, we discussed how ventilator models would benefit from providing a measure of uncertainty. In the future, demand calculators will ideally provide uncertainty in their estimates and be validated against robust usage ground truth data that also accounts for uncertainty.

We conclude with some recommendations based on our results and observations.

**Recommendation 1**: There should be efforts toward standardization in resource demand modeling.

As we explored the literature, and analyzed publicly available demand models, it was evident there is a need for standardization among terminology. Demand models varied in name ranging from burn rate calculator to epidemiological model to forecasting tool. We developed our own terminology (demand calculator) for this article. As this area of research continues to grow it will be important to have universal terminology to locate resource demand models and allow users to better understand differences in demand models. In addition to consistent terminology, the field would benefit if there was greater standardization of documentation and model/tool interface. While we do not propose standardization of modeling approaches, we found demand models difficult to locate, understand and in some cases run, due to the large differences in provided information. Standardization of terminology, documentation, interface, and other non-back-end aspects could increase visibility and usability.

**Recommendation 2**: Models should be made more transparent and accessible.

Demand models should be made more accessible to the public for visibility and transparency purposes. This can be done by making demand models open source with clear documentation of predictions. During our investigation of resource demand models, many models lacked substantial documentation. Some models that were accessible had undisclosed components or features hidden within Excel worksheets. Meanwhile other models had written documentation but an inaccessible tool. Publicly accessible models, with clear and comprehensive documentation on both the tool interface and modeling approach, would advance the field of medical resource demand modeling. A centralized hub for demand models, similar to [27] for COVID-19 epidemiological models, could be useful for promoting transparency and accessibility, as well as standardization (Recommendation 1). Moreover, multi-model hubs facilitate development of ensemble models, which can have better performance than component individual models.

**Recommendation 3**: There should be community-wide efforts towards collecting usage data that could be used for model validation.

There is also a clear need for validation data for medical resources utilized. Some data on medical resources data is available but not comprehensive (e.g., ventilators) whereas for other medical resources data is available but imperfect (e.g., testing kits). However, for most medical resources no data is available at all, especially for PPE needs. One nonprofit organization, Get Us PPE, collected data on requests for donated PPE from March 20, 2020 through July 2, 2021 [44]. Get Us PPE built up the largest non-governmental database on PPE shortages in the United States, which assisted in getting personal protective equipment to people who needed it most. This data was not sufficient for the needs of our analysis since it only captured when PPE needs were requested to this specific organization. Many PPE resources were sourced elsewhere, and thus this data set is not representative of the PPE demanded. PPE shortages were one of the largest concerns early in the pandemic, hence the number of PPE demand models. The reliability of these PPE models remains unclear due to the lack of validation data, especially given the wide range of model predictions seen in the Gowns section. In addition, greater and higher quality data can be used as training data and lead to improved models. Therefore, organizations and health care systems should make efforts to collect and make medical resource data available and accessible to the public.

**Recommendation 4**: A hierarchical validation framework for resource demand models should be developed.

We have provided a novel approach to evaluating demand models where we focused first on the epidemiological models and then validated the demand calculators using ground truth inputs. Once the above recommendations have been implemented this approach could be formalized into a hierarchical validation framework for demand models. The hierarchical approach would begin by validating the epidemiological model first, then the demand calculator with perfect inputs, and lastly analyze the full demand model. This would ultimately aid in the reliability of medical resource demand model performance and contribute to a better prepared medical resource supply chain.

**Recommendation 5**: The scope of the models should expand beyond the hospital setting.

To aid in medical resource preparedness in hopes to avoid medical device shortages, the scope of models should expand beyond a hospital setting. Currently, the demand models we examined only accounted for medical resource needs in hospitals and health care settings across the US. However, medical resources such as masks, testing kits, and hand sanitizer are not just necessary supplies within hospitals. Homes, schools, and businesses also need to be prepared with appropriate cleaning and safety measures. This would provide a more holistic picture of the true demand for medical resources needed by the public rather than solely capturing the healthcare setting. As a result, the medical supply chain can be better equipped during a public health emergency.

## Conclusions

The field of medical resource demand modeling is still in its infancy but has the potential to play a key role in optimizing resource allocation and preventing disastrous shortages in future public health emergencies. However, current models suffer from considerable uncertainty regarding their predictive capability, and the results of this paper suggest large errors are possible. The accuracy of these models is highly dependent on data quality, which can vary widely between healthcare systems and regions. Also, the modeling process often simplifies complex medical scenarios, potentially oversimplifying patient/staff needs or failing to capture the nuances of rapidly evolving healthcare landscapes. We believe that the continual advancements in modeling methods, computing capability and data availability, if supported by community efforts outlined in our recommendations, could bring the field of demand modeling to maturity as a vital resource for public health decision makers.

## Supplementary Information

Additional file 1: Assessing the reliability of medical resource demand models in the context of COVID-19. Includes figures of additional peak event model predictions, additional future cumulative hospital/admission model predictions for peak events, and state traces of the observed ratio of patients on ventilators to COVID-19 patients in the ICU.

## Data Availability

No datasets were generated or analysed during the current study.
