# Supplementary material for: Assessing the reliability of medical resource demand models in the context of COVID-19
Source: BMC Med Inform Decis Mak. 2024 Oct 31;24:322. doi: 10.1186/s12911-024-02726-6 (PMC11529025; doi:10.1186/s12911-024-02726-6)
Supplement: Supplementary file 1 — Additional file 1: Assessing the reliability of medical resource demand models in the context of COVID-19. Includes figures of additional peak event model predictions, additional future cumulative hospital/admission model predictions for peak events, and state traces of the observed ratio of patients on ventilators to COVID-19 patients in the ICU. [file 12911_2024_2726_MOESM1_ESM.pdf]

Additional file 1 for: Assessing the reliability of medical resource demand models in the context of COVID-19

1 Qualitative analysis of hospital use models

Figures S1–S7 provides every model's predictions for each peak event analyzed. The seven additional peak events analyzed are Florida, Georgia, Illinois, New York, Ohio, Pennsylvania, and Texas. Note that UCLA's model did not provide predictions for Florida, Georgia, and Ohio.

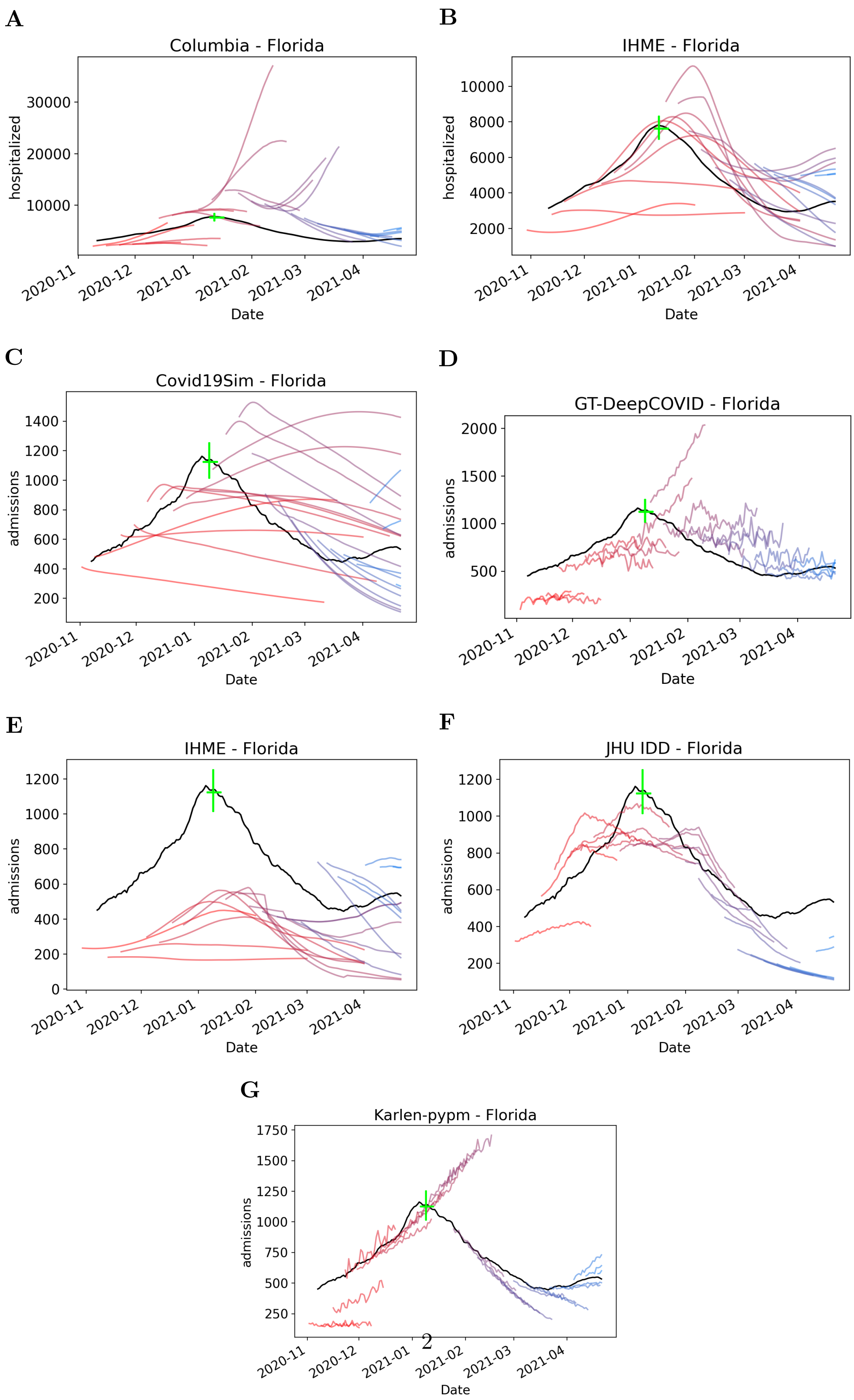

Fig. S1: Predictions for Florida during Fall 2020 for each model. Black line is the seven-day rolling average for recorded number of hospitalizations/admissions and green lines represent uncertainty in true peak date and magnitude. Red lines indicate early model releases; blue lines indicate later model releases; purple lines are intermediate results.

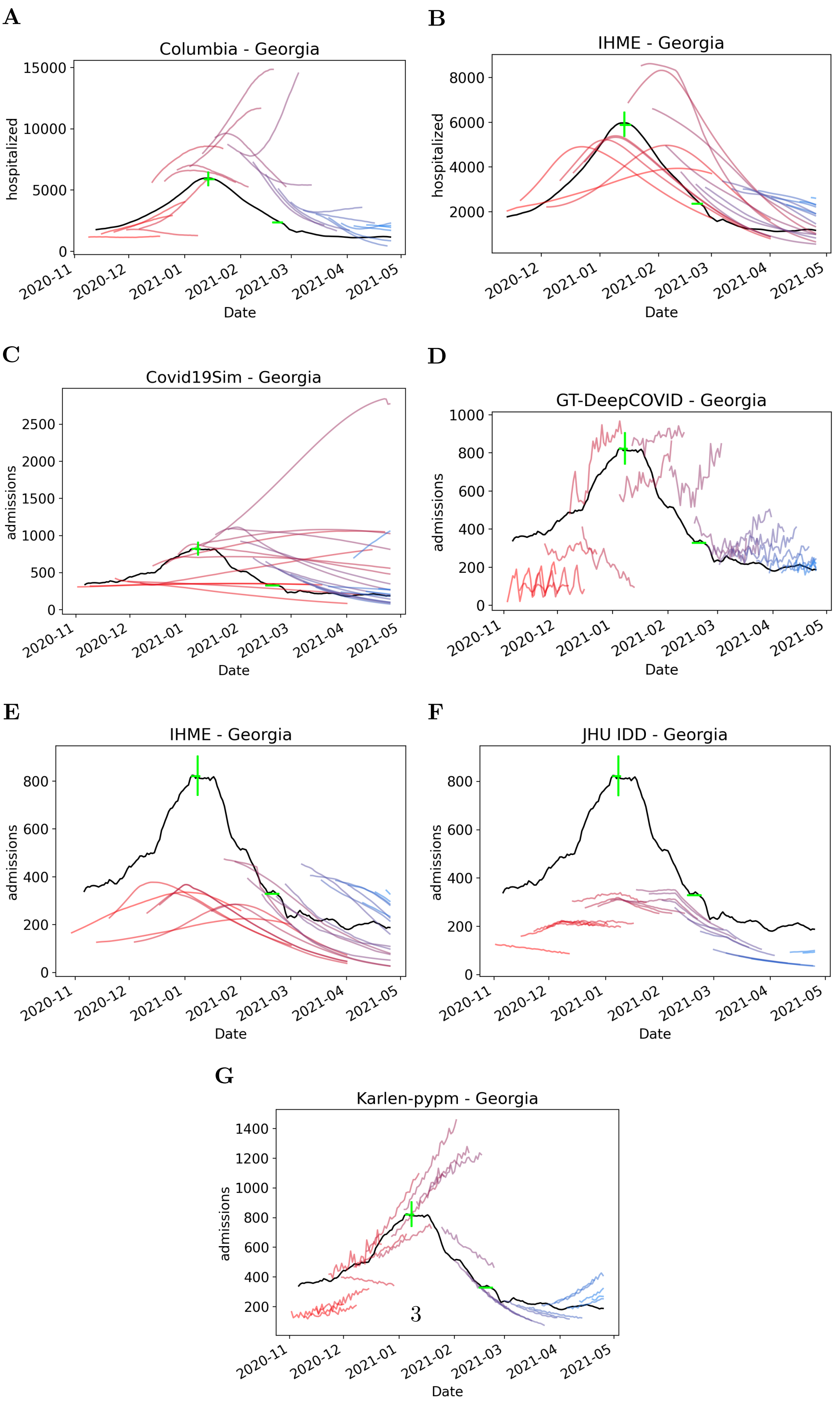

Fig. S2: Predictions for Georgia during Fall 2020 for each model. Black line is the seven-day rolling average for recorded number of hospitalizations/admissions and green lines represent uncertainty in true peak date and magnitude. Red lines indicate early model releases; blue lines indicate later model releases; purple lines are intermediate results.

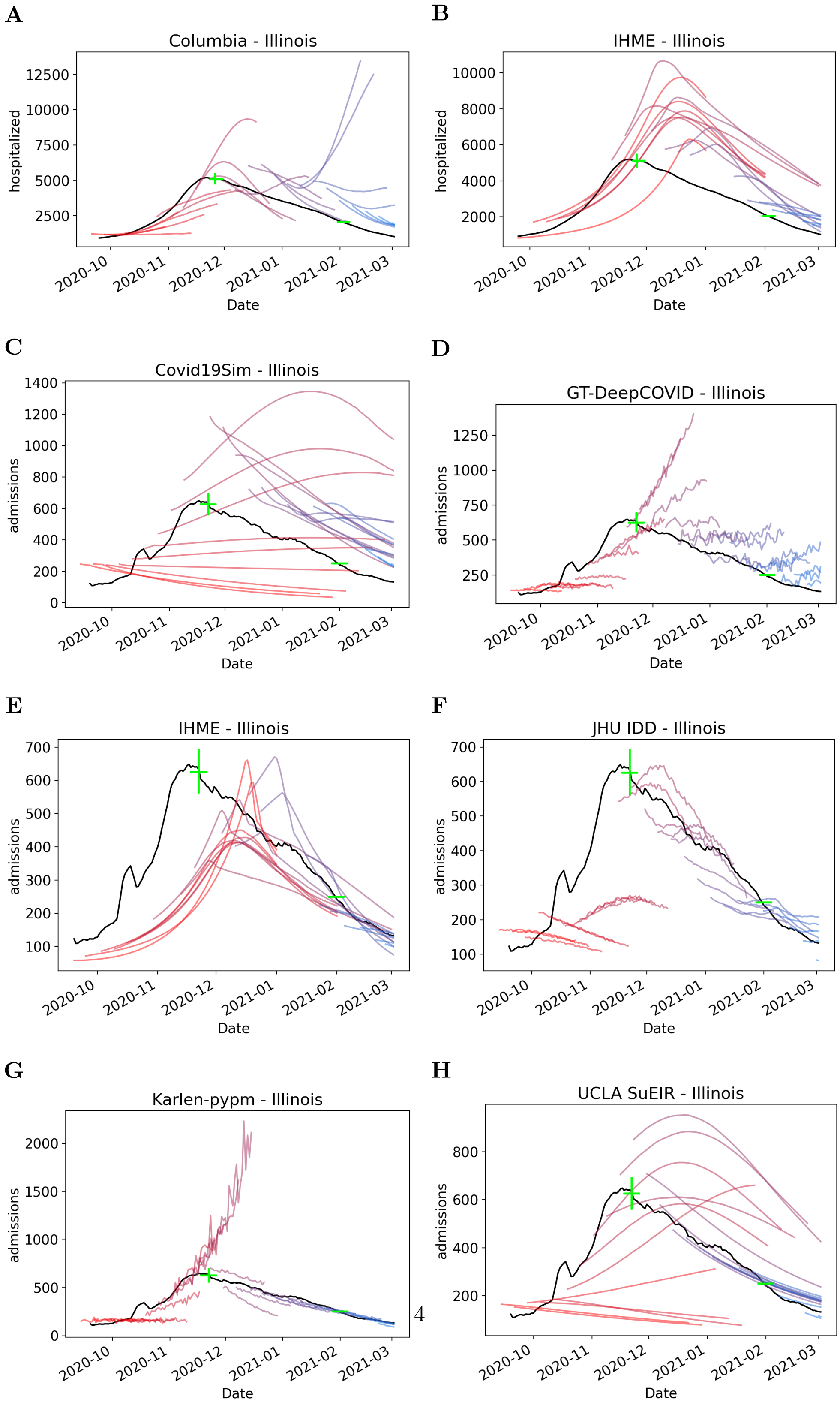

Fig. S3: Predictions for Illinois during Fall 2020 for each model. Black line is the seven-day rolling average for recorded number of hospitalizations/admissions and green lines represent uncertainty in true peak date and magnitude. Red lines indicate early model releases; blue lines indicate later model releases; purple lines are intermediate results.

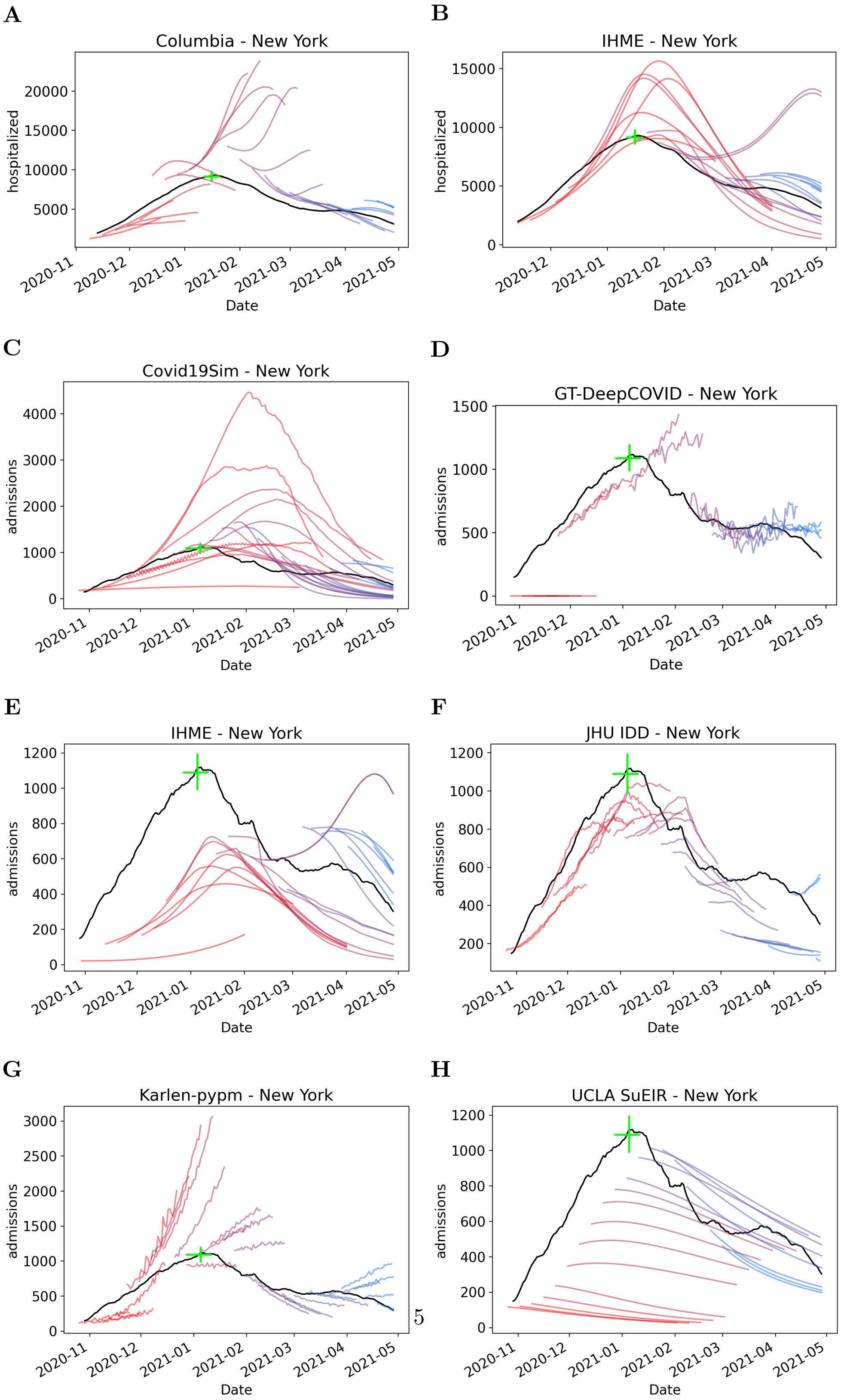

Fig. S4: Predictions for New York during Fall 2020 for each model. Black line is the seven-day rolling average for recorded number of hospitalizations/admissions and green lines represent uncertainty in true peak date and magnitude. Red lines indicate early model releases; blue lines indicate later model releases; purple lines are intermediate results.

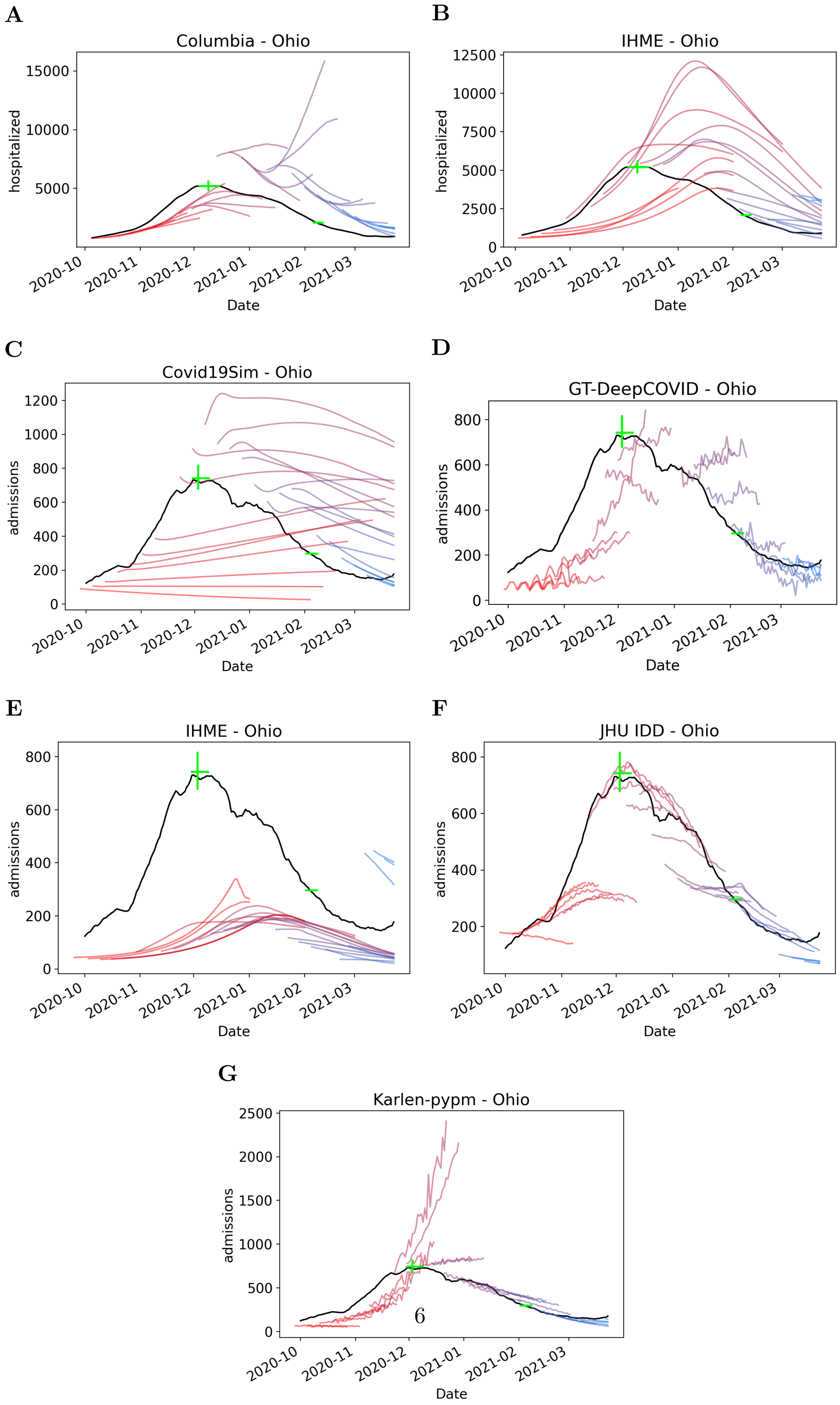

Fig. S5: Predictions for Ohio during Fall 2020 for each model. Black line is the seven-day rolling average for recorded number of hospitalizations/admissions and green lines represent uncertainty in true peak date and magnitude. Red lines indicate early model releases; blue lines indicate later model releases; purple lines are intermediate results.

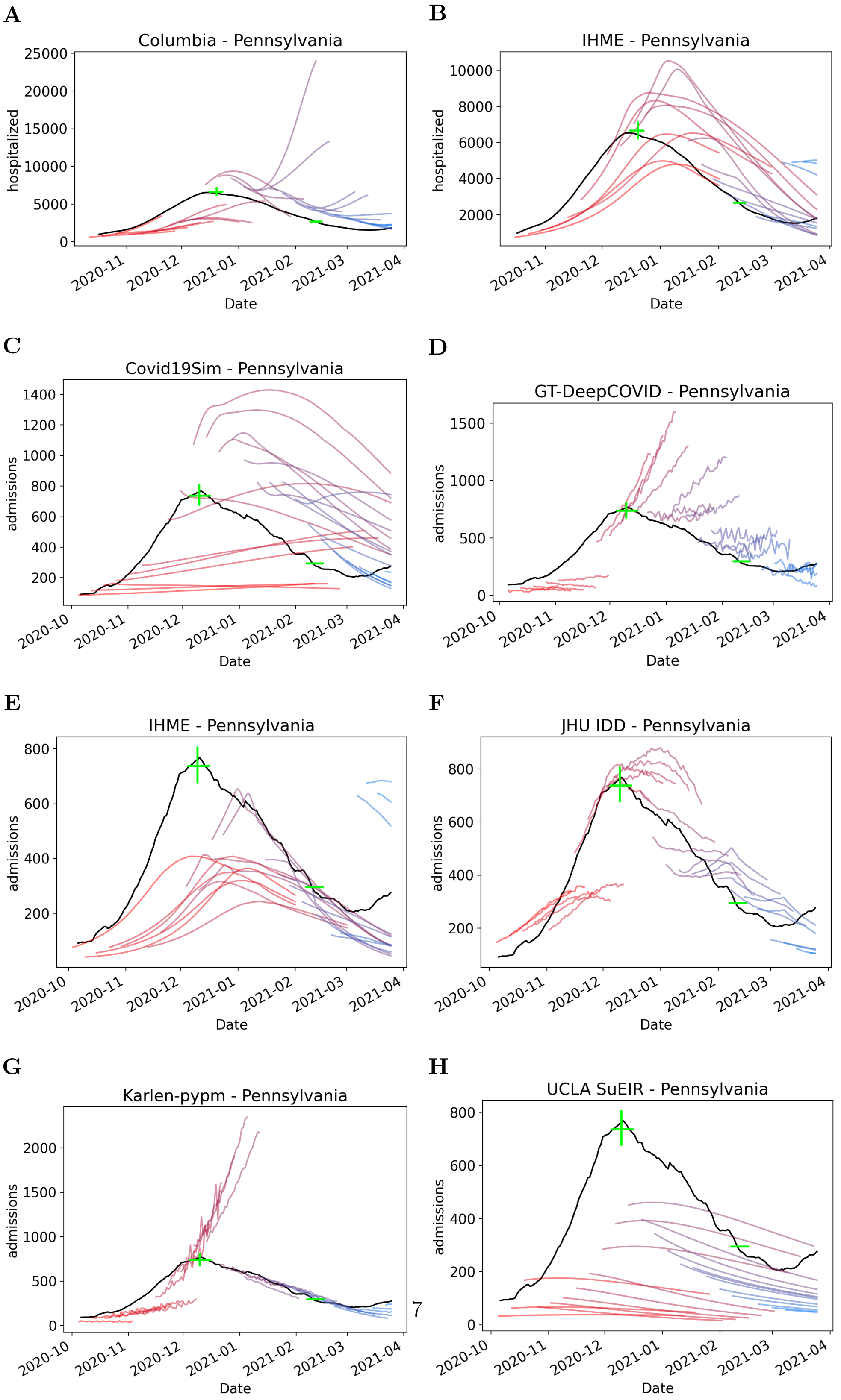

Fig. S6: Predictions for Pennsylvania during Fall 2020 for each model. Black line is the seven-day rolling average for recorded number of hospitalizations/admissions and green lines represent uncertainty in true peak date and magnitude. Red lines indicate early model releases; blue lines indicate later model releases; purple lines are intermediate results.

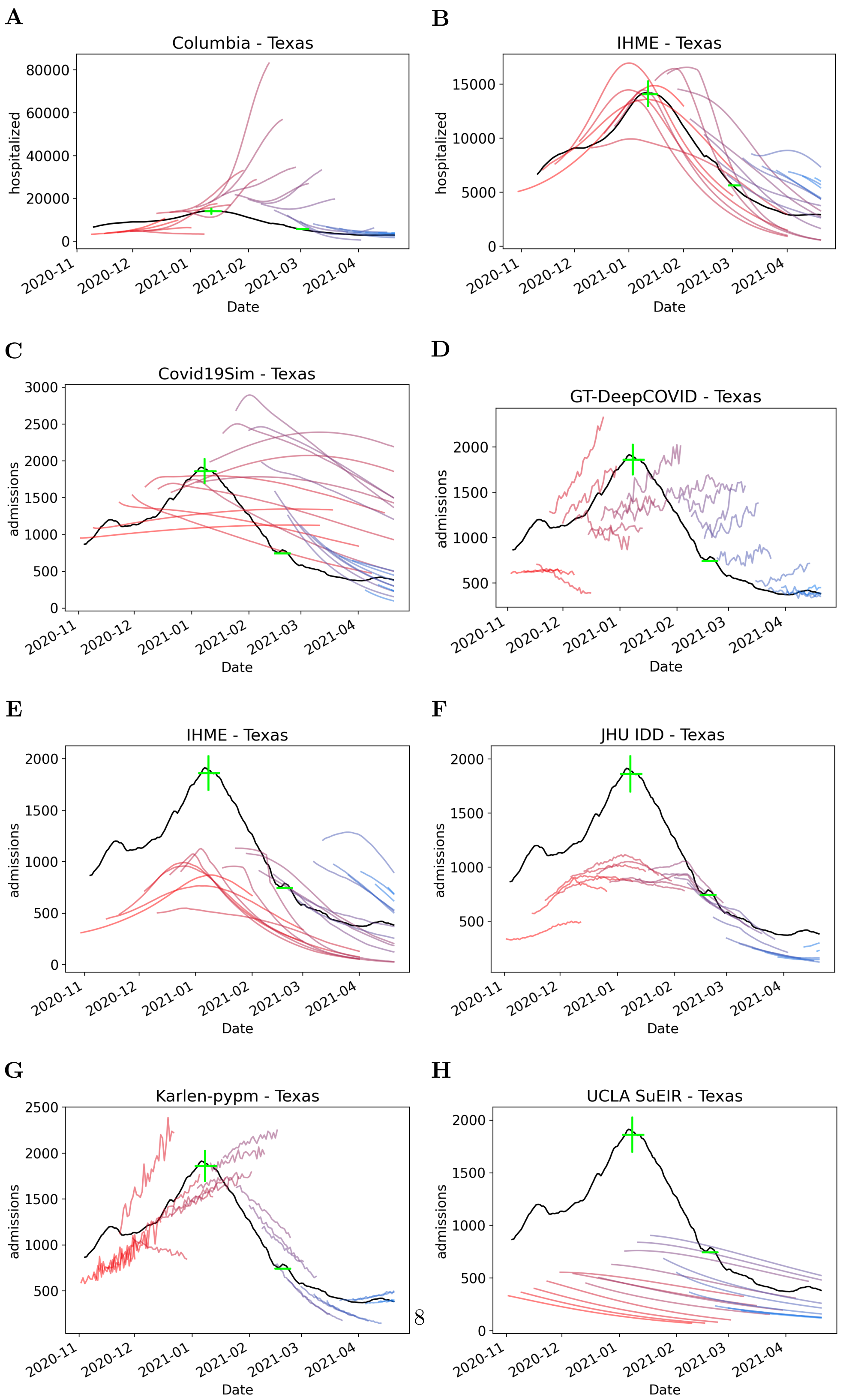

Fig. S7: Predictions for Texas during Fall 2020 for each model. Black line is the seven-day rolling average for recorded number of hospitalizations/admissions and green lines represent uncertainty in true peak date and magnitude. Red lines indicate early model releases; blue lines indicate later model releases; purple lines are intermediate results.

2 Accuracy of future cumulative hospitalizations/admissions predictions

Figures S8–S14 compares every model's future cumulative hospital/admission predictions to the observed cumulative hospitalizations/admissions for each peak event analyzed. Eight model's predictions were shared: Columbia, IHME (hospitalization), Covid19Sim, GT-DeepCOVID, IHME (admissions), JHU IDD, Karlen, and UCLA. The seven additional peak events analyzed are Florida, Georgia, Illinois, New York, Ohio, Pennsylvania, and Texas. Note that UCLA's model did not provide predictions for Florida, Georgia, and Ohio.

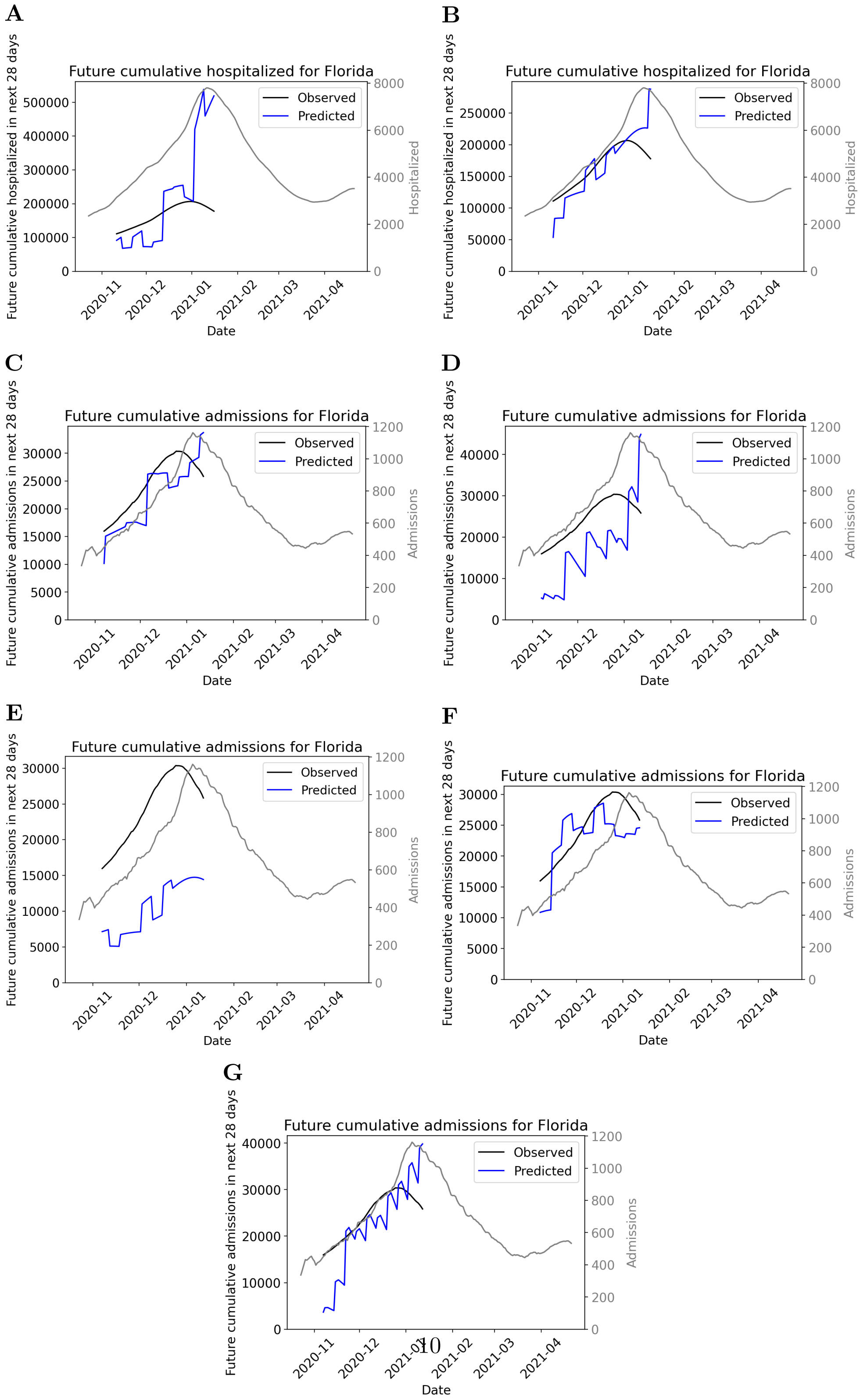

Fig. S8: The future cumulative hospitalization predictions over a 28-day period following a model release for A) Columbia and B) IHME; and future cumulative admission predictions over a 28-day period following a model release for C) Covid19Sim admissions, D) GT-DeepCOVID, E) IHME, F) JHU IDD, and G) Karlen, all for the Florida peak event. Blue line is model's predictions, black line is true cumulative hospitalizations/admissions data from HealthData.gov. Also shown for context is daily hospitalizations/admissions (grey line).

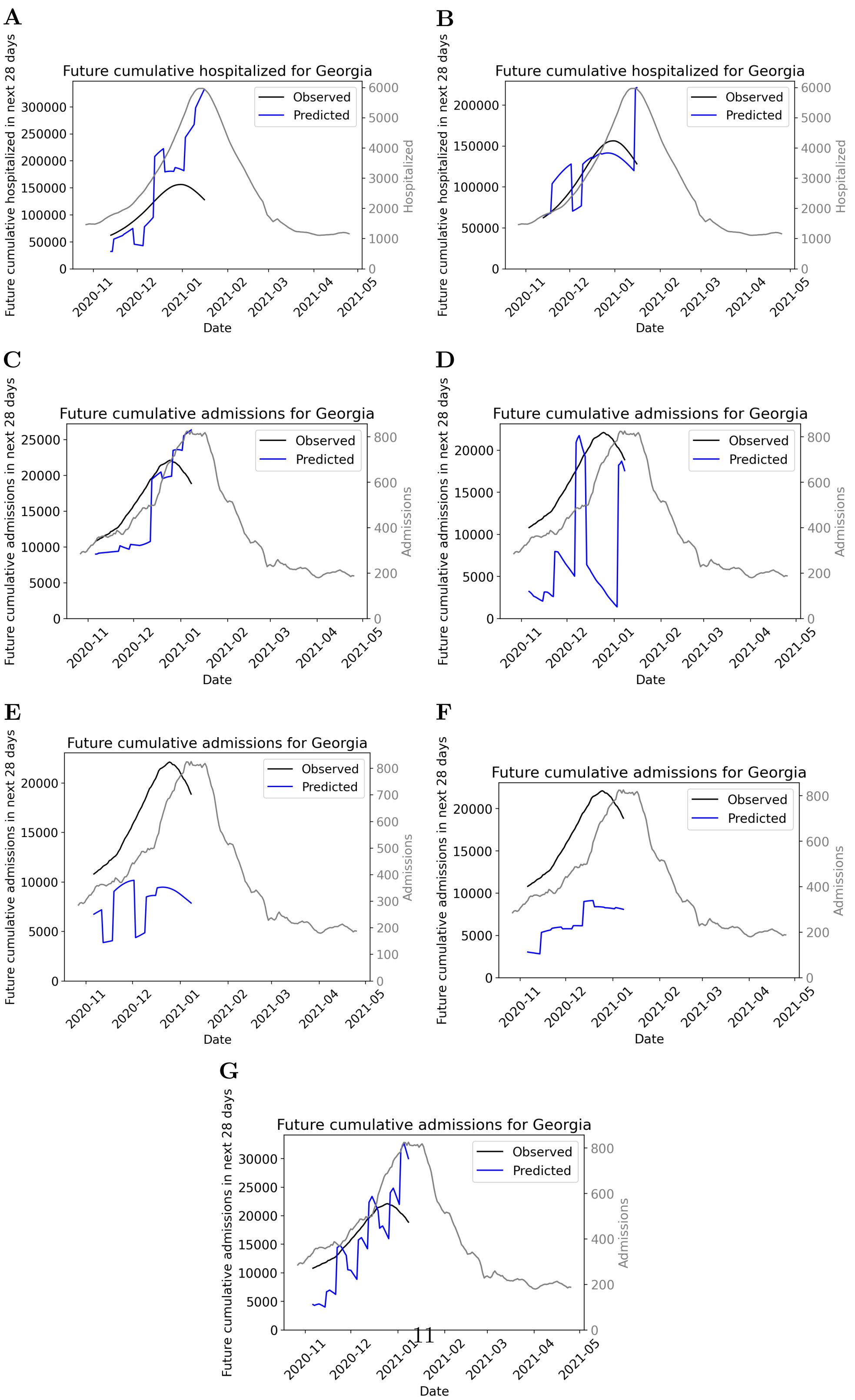

Fig. S9: The future cumulative hospitalization predictions over a 28-day period following a model release for A) Columbia and B) IHME; and future cumulative admission predictions over a 28-day period following a model release for C) Covid19Sim admissions, D) GT-DeepCOVID, E) IHME, F) JHU IDD, and G) Karlen, all for the Georgia peak event. Blue line is model's predictions, black line is true cumulative hospitalizations/admissions data from HealthData.gov. Also shown for context is daily hospitalizations/admissions (grey line).

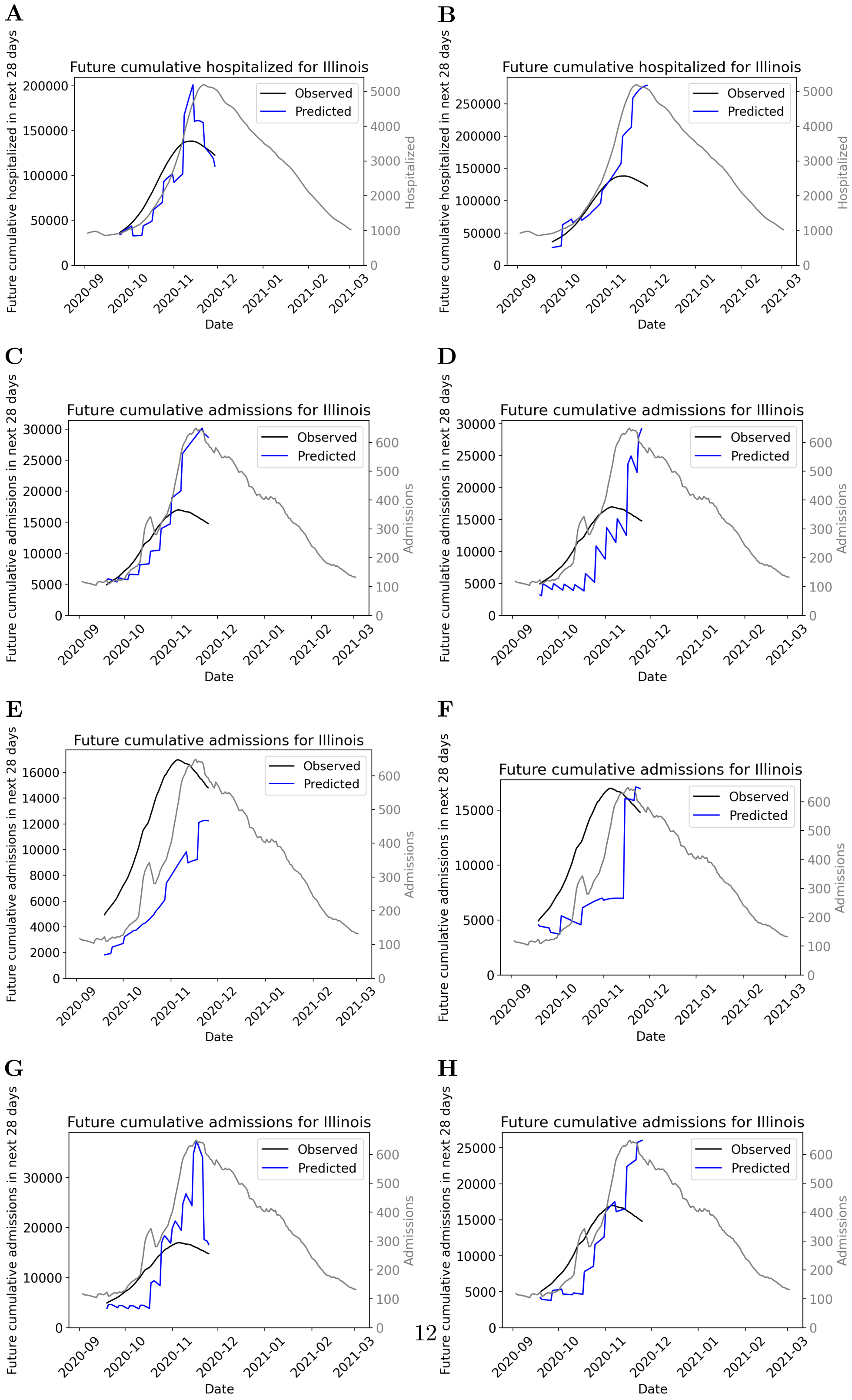

Fig. S10: The future cumulative hospitalization predictions over a 28-day period following a model release for A) Columbia and B) IHME; and future cumulative admission predictions over a 28-day period following a model release for C) Covid19Sim admissions, D) GT-DeepCOVID, E) IHME, F) JHU IDD, G) Karlen, and H) UCLA, all for the Illinois peak event. Blue line is model's predictions, black line is true cumulative hospitalizations/admissions data from HealthData.gov. Also shown for context is daily hospitalizations/admissions (grey line).

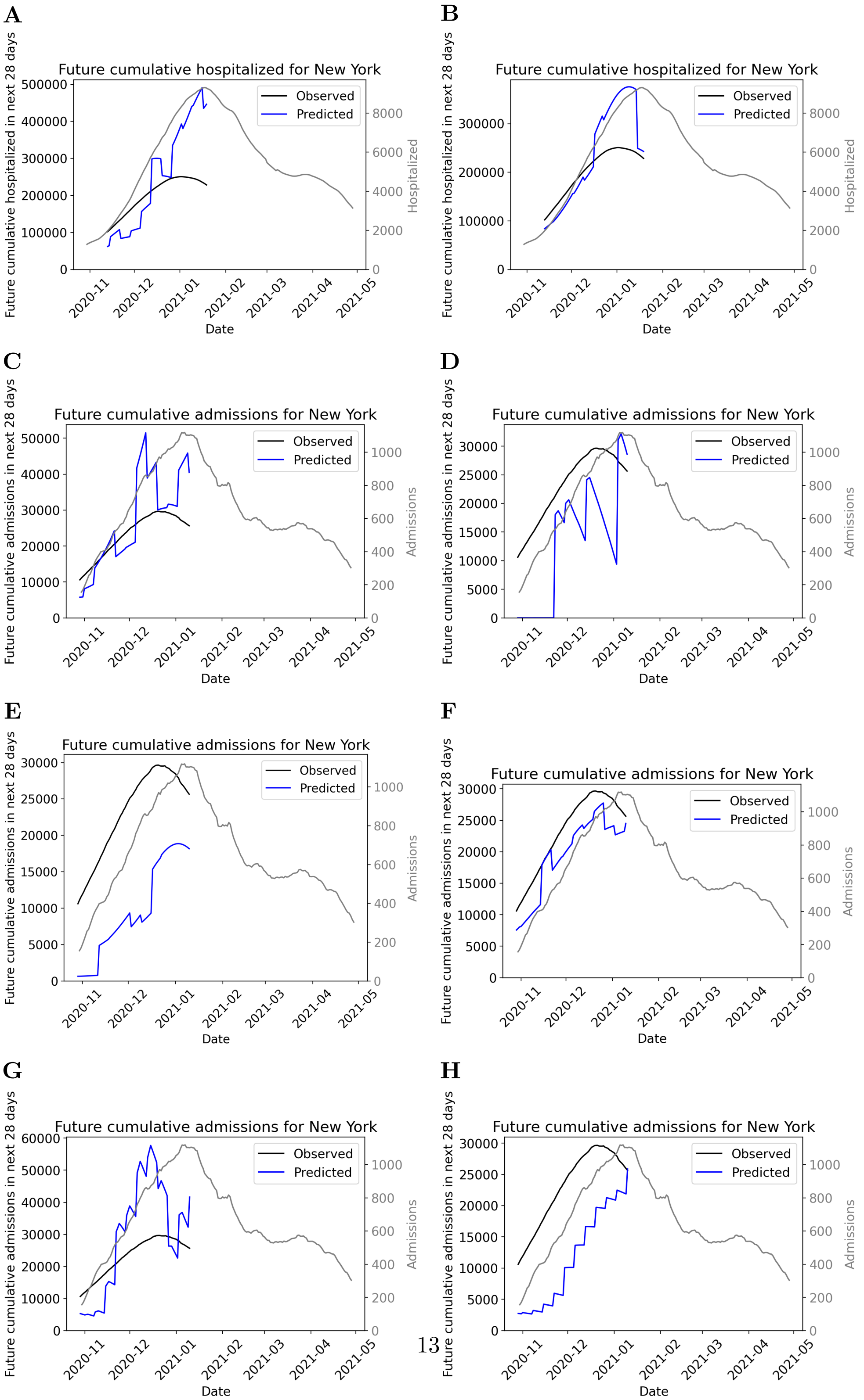

Fig. S11: The future cumulative hospitalization predictions over a 28-day period following a model release for A) Columbia and B) IHME; and future cumulative admission predictions over a 28-day period following a model release for C) Covid19Sim admissions, D) GT-DeepCOVID, E) IHME, F) JHU IDD, G) Karlen, and H) UCLA, all for the New York peak event. Blue line is model's predictions, black line is true cumulative hospitalizations/admissions data from HealthData.gov. Also shown for context is daily hospitalizations/admissions (grey line).

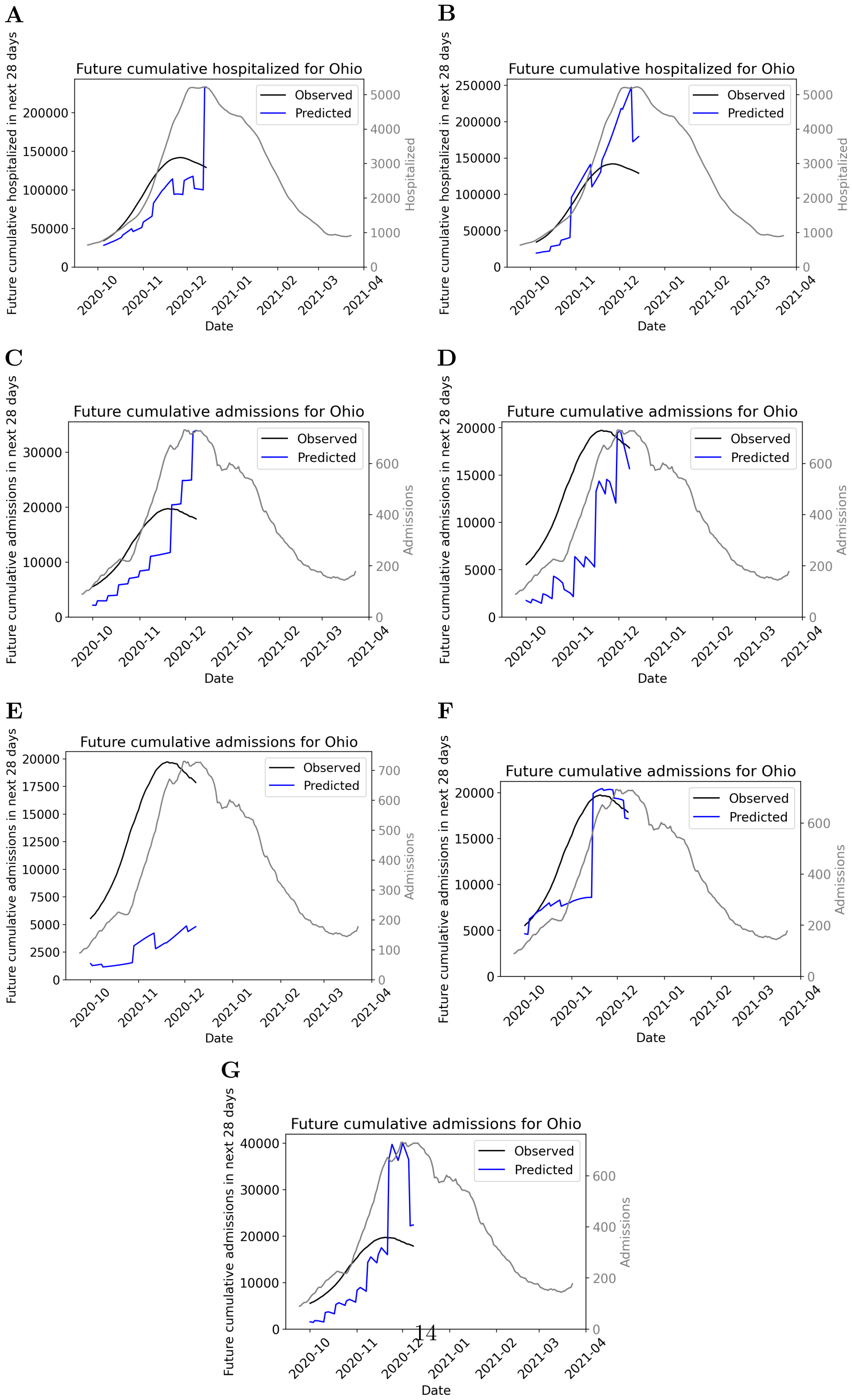

Fig. S12: The future cumulative hospitalization predictions over a 28-day period following a model release for A) Columbia and B) IHME; and future cumulative admission predictions over a 28-day period following a model release for C) Covid19Sim admissions, D) GT-DeepCOVID, E) IHME, F) JHU IDD, and G) Karlen, all for the Ohio peak event. Blue line is model's predictions, black line is true cumulative hospitalizations/admissions data from HealthData.gov. Also shown for context is daily hospitalizations/admissions (grey line).

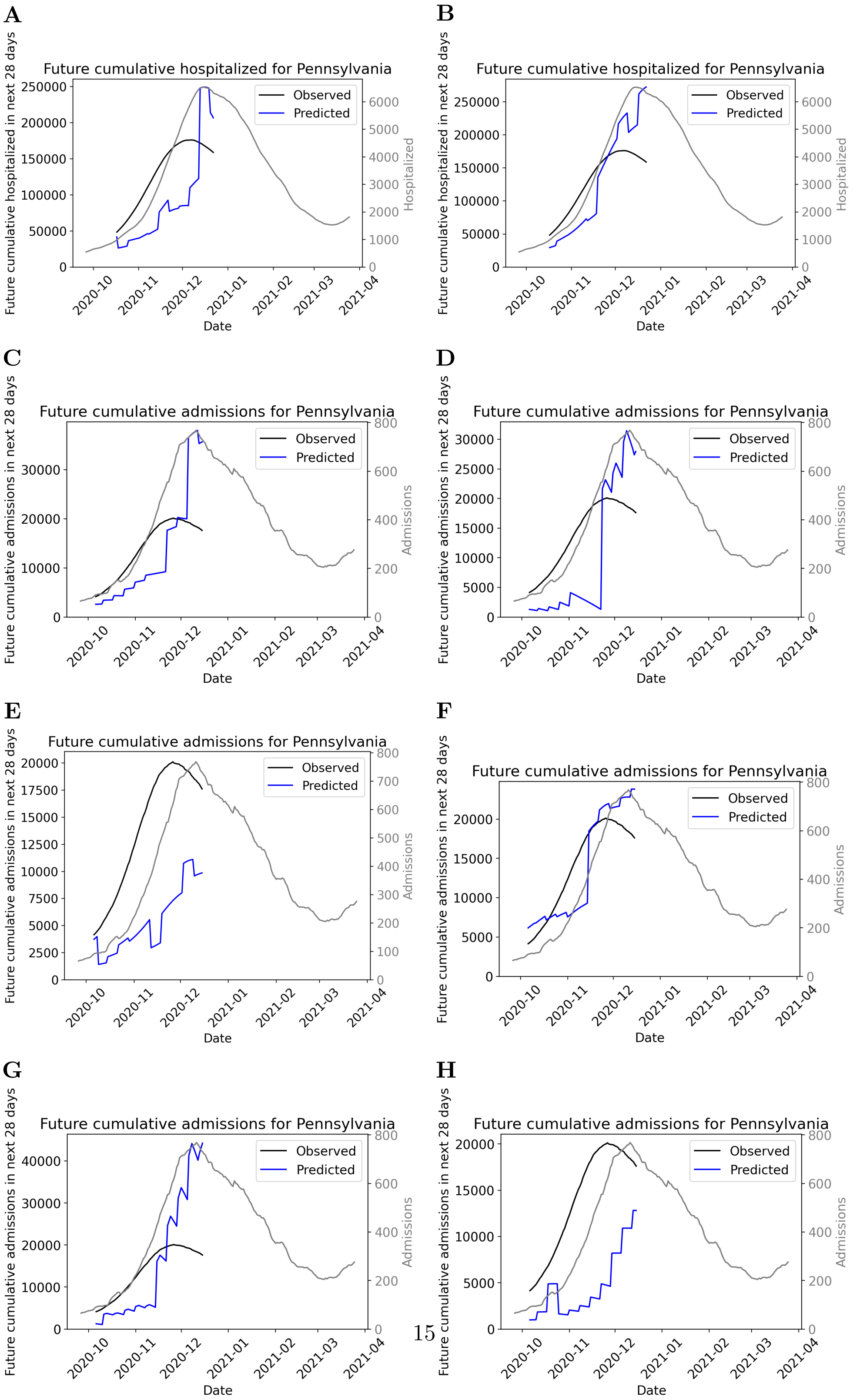

Fig. S13: The future cumulative hospitalization predictions over a 28-day period following a model release for A) Columbia and B) IHME; and future cumulative admission predictions over a 28-day period following a model release for C) Covid19Sim admissions, D) GT-DeepCOVID, E) IHME, F) JHU IDD, G) Karlen, and H) UCLA, all for the Pennsylvania peak event. Blue line is model's predictions, black line is true cumulative hospitalizations/admissions data from HealthData.gov. Also shown for context is daily hospitalizations/admissions (grey line).

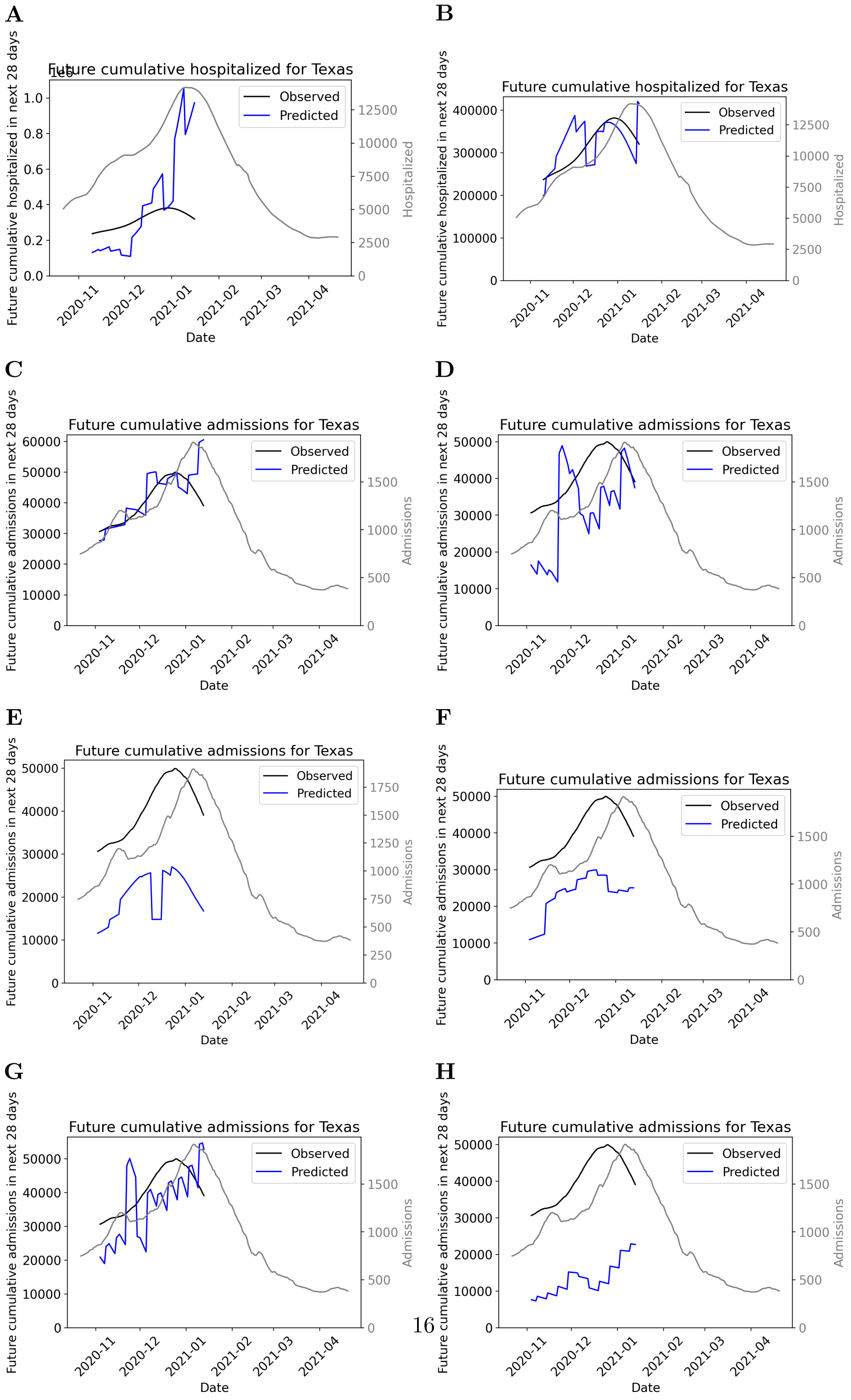

Fig. S14: The future cumulative hospitalization predictions over a 28-day period following a model release for A) Columbia and B) IHME; and future cumulative admission predictions over a 28-day period following a model release for C) Covid19Sim admissions, D) GT-DeepCOVID, E) IHME, F) JHU IDD, G) Karlen, and H) UCLA, all for the Texas peak event. Blue line is model's predictions, black line is true cumulative hospitalizations/admissions data from HealthData.gov. Also shown for context is daily hospitalizations/admissions (grey line).

3 Ventilators

Figure S15 below displays variations of Figure 7 in the manuscript, containing traces of the observed ratio of patients on ventilators to COVID-19 patients in the ICU for a small number of states per figure, so that individual traces can be discerned.

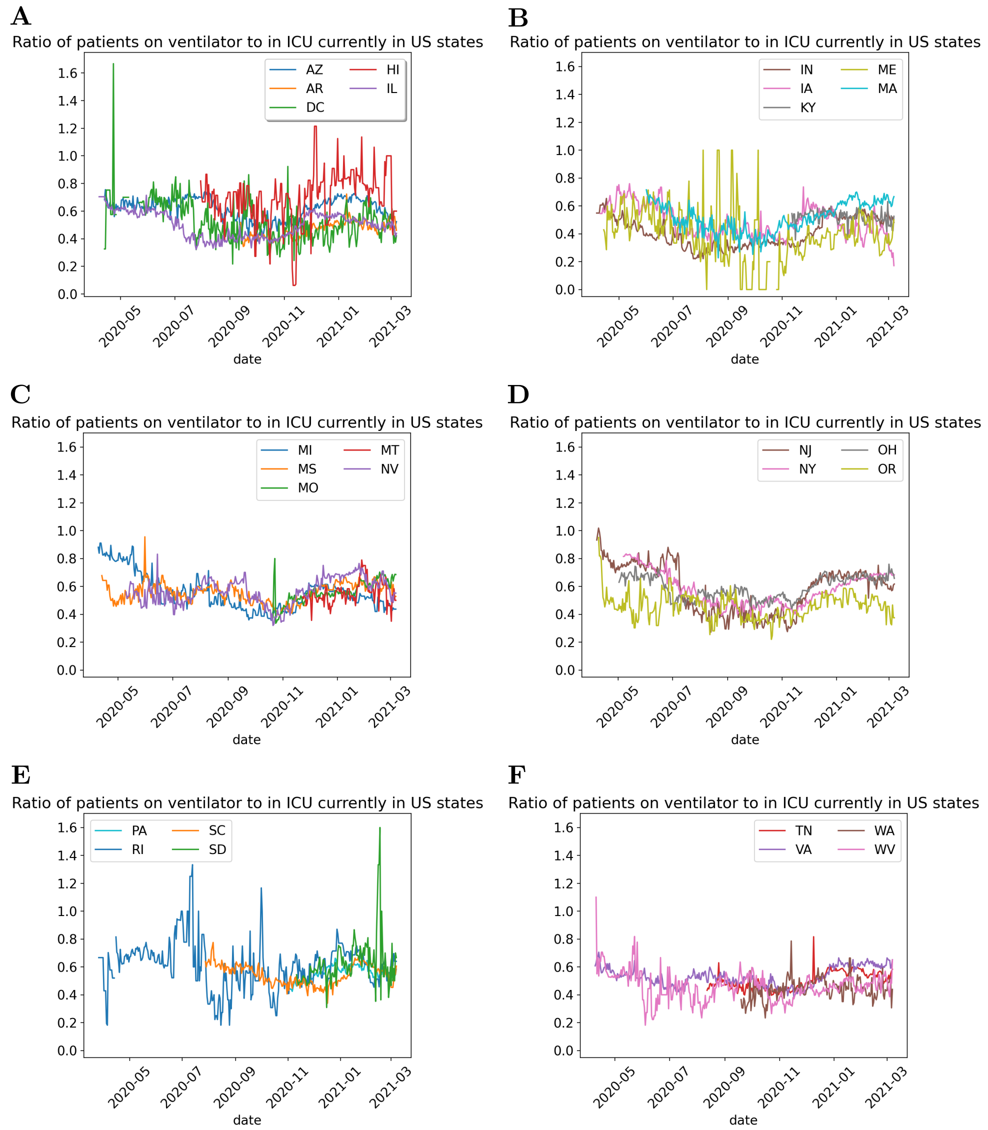

Fig. S15: Ratio of number of patients on ventilator to number of patients in the ICU. Various colored lines represent ratio for different states from April 2020 to March 2021.
